# Gut Microbiota and Serum Metabolite Potential Interactions in Growing Layer Hens Exposed to High-Ambient Temperature

**DOI:** 10.3389/fnut.2022.877975

**Published:** 2022-04-27

**Authors:** Changming Zhou, Xiaona Gao, Xianhong Cao, Guanming Tian, Cheng Huang, Lianying Guo, Yulan Zhao, Guoliang Hu, Ping Liu, Xiaoquan Guo

**Affiliations:** Jiangxi Provincial Key Laboratory for Animal Health, College of Animal Science and Technology, Jiangxi Agricultural University, Nanchang, China

**Keywords:** global warming, heat stress, gut microbiota, metabolomics, correlation analysis, layer hens

## Abstract

Emerging evidence has revealed the dysbiosis of gut microbiota contributes to development of metabolic diseases in animals. However, the potential interaction between gut microbiota and host metabolism in growing hens under metabolic disorder induced by chronic heat exposure (CHE) remains inconclusive. The aim of our study was to examine the potential association among the cecal microbiota community, physiological indicators, and serum metabolite profiles in CHE hens. One hundred and eighty Hy-Line Brown hens were randomly allocated into three groups: thermoneutral control (TN), heat stress (HS), and pair-fed (PF). The experiment lasted for 5 weeks, with the first 2 weeks serving as the adaptation period. Results showed that the expression level of heat shock protein 70 (HSP70) in both serum and cecal tissues was significantly increased in the HS group. Serum parameters analysis also revealed that CHE caused physiological function damage and metabolic disorders. These results suggest the experiment was successful, inducing chronic heat stress. 16S rRNA sequencing analysis showed that the CHE can clearly induce dysbiosis of the gut microbial community reflected in the increment of the F/B ratio. Besides, serum untargeted metabolomics revealed the relative concentrations of 40 metabolites were significantly altered in the HS group compared with the TN group. Pathway analysis showed that these metabolites were mainly involving the increased proteolysis rather than lipolysis, and this tendency could be a specific metabolic adaptation of the poultry. The pair-feed experiment showed that the above changes induced by CHE were partly independent from the reduction of feed intake. Mantel correlation analysis between gut microorganisms and physiological indicators showed that the phylum *Firmicutes* and *Euryarchaeota* have a potential interaction with a serum lipid parameter. Random forest analysis showed that both genus *Faecalibacterium* and *Methanobrevibacter* were important predictors of the CHE-induced lipid metabolism disorder. Taken together, our findings may contribute to a better understanding of the metabolic mechanisms underlying the energy metabolism imbalance caused by the CHE and provide novel insights into the host-microbes interactions and its effects on the metabolic adaptation of hens under chronic heat exposure.

## Introduction

Given the increasing frequency, duration, and intensity of heat waves because of global warming, high-ambient temperature-induced heat stress (HS) has created a massive challenge for poultry production system (1). Simultaneously, the global demand for poultry meat and eggs is continuously increasing in parallel to rising population, urbanization, and an improved standard of living in developing countries (2, 3). In response to this demand, the poultry industries made dramatic improvements in meat, egg production, and feed efficiency through the genetic selection, nutrition improvement, husbandry, management, and vertical integration during the past few decades (4, 5). However, as adopting high-stocking densities and lack of genetic selection for resistance to extreme temperatures, the modern poultry farming is more sensitive to high temperatures than ever before (6). Actually, the domestic birds, unlike mammals, are lack of sweat glands and insulating feathers hampered heat dissipation, leading it naturally prone to heat stress (7, 8). Besides, reduced growth performance, productivity, nutrient utilization, and increased mortality caused by HS have been well documented, which makes enormous economic losses annually to the poultry industry (9, 10).

A growing number of studies implicate the chronic heat exposure (CHE) not only has detrimental effects on physiological, immunological, reproductive, and gut status of poultry birds but also leads to dysregulation of energy balance and metabolism (11). For instance, the respiratory rate raised in broilers during high climatic temperature in order to increase heat loss, which further increases the maintenance energy requirement for fulfillment of body physiological demand, combined with the reduction of feed intake, ultimately induces glycogen depletion and negative energy balance (NEB) (12). When animals are in a severe NEB state, suffer intensified catabolism of fat and protein, resulting in energy store mobilization (13, 14). However, contradictory findings have been reported that heat-exposed chickens exhibit enhanced fat deposition, which finally affects carcass characteristics (15, 16). Another related study suggested that, as the energetic efficiency of lipid deposition is higher than protein deposition, the CHE-induced lipid accumulation might be an adaptive response to reduce metabolic heat production in birds (16, 17). Supportive of this, findings reported by Temim et al. show that the CHE can inhibit muscle protein deposition in chickens mainly through reducing protein synthesis (18). However, the underlying mechanism by which chronic heat exposure leads to fat accumulation in chickens is still unclear. Nowadays, metabolomics has become a powerful systematic biological analysis tool that can identify and quantify the global changes of disease-specific endogenous small-molecule metabolites in a biological sample using high-throughput techniques, and allows for a wide understanding of the biological information associated with metabolites (19). Recently, untargeted serum metabolomics study has revealed that, when confronted with a negative energy balance state induced by CHE, broilers failed to effectively mobilize body fat, instead of resorting to protein decomposition for their energy production (12). Of note, previous studies have proposed that the adverse impact of heat stress on fat deposition depends on the breed types of chickens (20). Similar results were obtained by Liao et al., who observed that the metabolic adaptations to heat stress in three beef cattle breeds are heterogeneous by using metabolomics profiling of serum and urine (21). Despite extensive pieces of research investigating the change of fat deposition and the metabolic adaptation of the heat-stressed broiler chickens, little has been researched on layer chickens.

Gut microbiota is now increasingly recognized as a hidden “metabolic organ” that plays a critical role in regulating the host metabolic homeostasis and energy balance, promoting the development and maturation of intestinal mucosa, maintaining intestinal barrier integrity in gut (22, 23). A normal gut microbial community and complete mucosal morphology in birds are the bases for excluding pathogens and maintaining normal nutrient digestion and absorption (24, 25). Cumulating evidence shows that heat stress redistributes blood flow in birds to the periphery for increased heat dissipation, which reduces oxygen and nutrient supply to intestinal tissues (26, 27). This adaptive change can cause damage to intestinal barrier functions during chronic heat exposure, which further affects metabolic activities in heat-stressed chickens through facilitating translocations of bacteria and endotoxins from the intestine to the circulation (8, 28). Recent studies have found gut microbiota made great contributions to fat deposition in chickens (29, 30). He et al. also detected that HS-induced gut microbiota dysbiosis may have potential relationships with the abnormal fat deposition in ducks (25). Besides, gut microbiota is highly sensitive to the early environment stress because the colonization of gut microbiota during the early life of animals is a time of significant fluctuation and maturation (31, 32). Previous studies have shown the alterations in the normal succession of gut microbiota in early life impact on future colonization, and the initial development of gut microbiota also has a long-term physiological effect on the host (32, 33). Whether exposed to heat-ambient temperature in hens during the growing period affects the colonization and functional maturation of intestinal microbiota and changes the metabolism between the host and intestinal microbiota remains unknown. Thus, it is necessary and meaningful to examine the altered microbe and metabolism pathways linked to growing hens during a prolonged period of heat exposure.

Here, we established a CHE model of growing hens to identify the influence of heat stress induced by chronic exposure to high environmental temperature on hens during the growing period. Another goal of this study was to examine the effects of CHE on the physiological indicators and describe the serum metabolic profile and alterations of cecal microbiota in heat-stressed hens and provide a deeper understanding of potential interaction between its altered physiological indicators/serum metabolites and cecal bacteria. For this end, we first assessed changes in physiological, histological, and biochemical parameters of the CHE hens. Then, we used an integrated approach comprising 16S rDNA sequencing and ultra-high performance liquid chromatography, coupled with quadrupole time-of-flight mass spectrometry (UPLC-Q-TOF/MS) to analyze the alterations in the hen cecal microbiome and a serum metabolic profile. The mantel test was conducted to find out the potential interaction pattern between cecal microbiota (phylum) and physical indicators in the CHE hens. Using random forest regression modeling (RF) and spearman correlations analysis, we further checked the contributions and interaction pattern of the relative abundances of distinct genera in cecal microbiota on the variation of serum metabolic profiles. The results would contribute to a better understanding of the metabolic mechanisms underlying the energy metabolism imbalance caused by the CHE and provide novel insights into the host-microbes interactions and its effects on the metabolic adaptation of hens under chronic heat exposure.

## Materials and Methods

### Animals and Sample Collection

All experimental procedures involving animals used in this study were conducted according to the guide for the care and use of laboratory animals of the National Institutes of Health, and the animal protocol was reviewed and approved by the Animal Care and Use Committee of Jiangxi Agricultural University (permit No. JXAULL-2020-28). All efforts were made to minimize animal suffering and to reduce the number of animals used. One hundred and eighty Hy-Line Brown hens were gotten from a commercial at 10 weeks of age (Guohua Co. Ltd., Nanchang, China). After 2 weeks of acclimation, the birds weighed an average of 1,103 ± 27 g and were randomly assigned into three groups, each group of birds was further subdivided into 6 replicate groups (10 chickens per replicate): TN (the thermoneutral control group, maintained at 22 ± 1°C for 24 h/d and *ad libitum*), HS [the heat stress group, maintained at 22 ± 1°C for 14 h/d (18:00 to 08:00) then 36 ± 1°C for 10 h/d (08:00 to 18:00) and *ad libitum*], PF (the pair-fed group, maintained at 22 ± 1°C for 24 h/d and fed the same amount as the HS group). We set the PF group to determine whether the effects of heat stress on the serum metabolic profiles and gut microbiota are independent of the reduction in feed intake (Supplementary Figure S1). During the 3-week experimental period, all birds received the basal diet (Supplementary Table S1) formulated to meet nutrient recommendations according to National Research Council (NRC) (2012). The relative humidity was controlled at 55 ± 5%, and a regular 12-h light–dark cycle was established.

**Figure 1:**
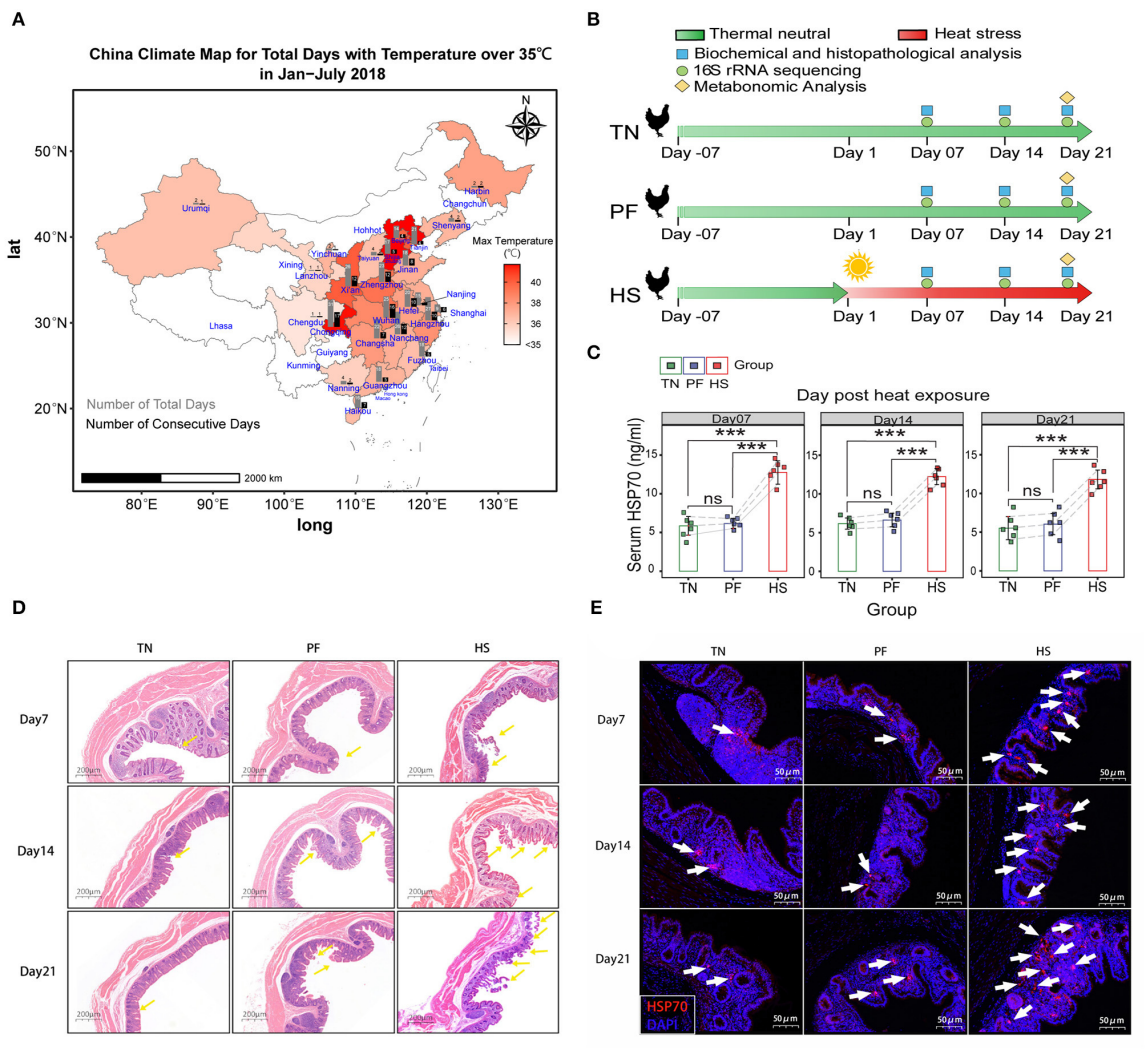
Establishment of chronic heat exposure models of growing hens. **(A)** China Climate Map for Total Days with Temperature over 35°C in January–July 2018. Colors represent the max temperatures among different provinces shown in the scale bar next to the charts. The gray bars represent the number of the total days, and black bars represent the number of consecutive days. **(B)** An overview of the schematic diagram for the time design of the experiment, before heat exposure (day-14), beginning of the heat exposure (Day 1), sampling days 7, 14, and 21. **(C)** Bar plots showing the level of HSP70 in serum among the TN, PF, and HS groups. All results are expressed as mean ± SD of eight hens in each group. *, **, and *** represent statistical significance at *p* < 0.05, *p* < 0.01, and, *p* < 0.001 levels. ns, denotes no significant difference was found in related groups. **(D)** Representative hematoxylin–eosin-stained images of (original magnification, 50×) of hen cecal tissue at different time points. **(E)** Representative images of immunofluorescence staining for HSP70 in cecal tissues.

The timeline of the experimental bird treatment and sample collection is shown in Figure 1B. Six individuals in each group were randomly sacrificed and euthanized on the 7th, 14th, and 21st days. On collection, the blood samples were collected from the external jugular vein into anticoagulant (EDTA) tubes (2 ml) at 2,500 rpm for 15 min at 4°C, and then serum was separated and stored at 20°C for a subsequent study. The luminal cecum contents were immediately frozen in liquid nitrogen and stored at −80°C until analysis. Meanwhile, the cecal tissues were prepared for histopathological and immunofluorescence analysis.

### Measurements of Serum Parameters

For serum biochemical assessment, the total protein (TP), albumin (ALB), globulin (GLO), total bilirubin (TBIL), direct bilirubin (D-BIL), indirect bilirubin (I-BIL), creatinine (CREA-S), uric acid (UA), total cholesterol (TC), triglycerides (TG), high-density lipoprotein (HDL), low-density lipoprotein (LDL), Alanine transaminase (ALT), aspartate transaminase (AST), alkaline phosphatase (ALP), γ-glutamyl transferase (γ-GT), lactate dehydrogenase (LDH), creatine kinase (CK), and myocardial creatine kinase (CK-MB) were evaluated using an automatic analyzer (Hitachi7060, Hitachi, Tokyo, Japan). For determination of antioxidant parameters, the total antioxidant capacity (T-AOC, kit number: A015-1-1), superoxide dismutase (SOD, kit number: A001-1-1), catalase (CAT), glutathione peroxidase (GSH-Px, kit number: A005-1-1), nitric oxide (NO, kit number: A012-1-2), and malondialdehyde (MDA, kit number: A003-1-1) levels in serum were measured using respective assay kits (Nanjing Jiancheng Bioengineering Institute, Nanjing, China). The levels of heat shock protein (HSP70) in serum were detected using a commercial ELISA kit (Chicken Heat Shock Protein 70, CSBE11196Ch, Cusabio, Wuhan, China). All experimental procedures were performed according to the manufacturer's recommended protocol.

### Pathological and Immunofluorescence Analysis

Histology was performed after prolonged heat exposure. Briefly, when animals were sacrificed, freshly harvested ceca tissues rapidly fixed in 10% phosphate-buffered formalin acetate at 4°C overnight, followed by dehydration in a series of graded ethanol, cleared with ultra-pure acetone, and then embedded in paraffin (a BMJ-III embedding machine, Jiangsu, China) wax at 56°C. Next, the wax blocks were cut into 5-μm-thick sections, stained with hematoxylin and eosin stain for inspection under a light microscope (Olympus, Tokyo, Japan) of any histopathological alterations. To examine the distribution of HSP70 protein in the cecal tissues from the three experimental groups of hens, we conducted immunofluorescence staining according to the method described in our previous study (34). The paraffin-embedded 5 μm-thick sections were deparaffinized, rehydrated, and then subjected to endogenous peroxidase blockage in 3% H_2_O_2_ and antigen retrieval in boiling a 10% citrate buffer. Subsequently, the sections were blocked with 3% normal goat serum in 0.1% Triton-X100 in PBS for 1.5 h, and then were incubated overnight at 4°C with primary antibodies (a Mouse Anti-HSP70 antibody (BB70) ab53496, 1:200, Abcam, Cambridge, MA, USA). After rinsing three times with PBS, the sections were treated by the goat anti-rabbit IgG-Cy3 secondary antibody (Servicebio Technology Co., Ltd., Wuhan, China), and then incubated at room temperature in the dark for 50 min. Following three washes with PBS, the sections were stained with 0.1 g/ml 4′-6-diamidino 2-phenylindole (DAPI, Servicebio Technology Co., Ltd., Wuhan, China) at room temperature for 5 min in the dark to identify nuclei. Finally, the stained sections were examined under a laser scanning confocal microscope (NIKON A1R, Nikon Corp., Tokyo, Japan).

### DNA Extraction and Illumina High-Throughput Sequencing of 16S RRNA Gene Sequence

Total microbial genomic DNA in the cecal content of chickens was extracted and then sent to BGI Co., Ltd (Wuhan, China) to prepare a 16S sequencing library for amplicon high-throughput sequencing according to the standard Illumina protocol (16S Metagenomic Sequencing Library Preparation). The amplification of the V4 region (515–806) of the 16S rRNA gene was conducted with a common primer pair (515F 5′-GTGCCAGCMGCCGCGGTAA-3′ and 806R 5′-GGACTACHVGGGTWTCTAAT-3′) (35). Then, purified amplicons were pooled in equimolar ratios and paired-end sequenced (2 × 250) on an Illumina HiSeq platform (Illumina, Inc., San Diego, CA, USA), following the manufacturer's guidelines. All the sequences in the present study were deposited in the NCBI Sequence Read Archive (SRA) database under accession no. PRJNA797199. The bioinformatics analysis was performed according to the standard protocols of BGI Tech Solutions Co., Ltd. Details of wet lab procedures and bioinformatic analysis are provided in the Supplementary Materials.

### UPLC-Q-TOF-MS Analysis of Metabolites in the Serum

Serum samples were prepared according to our previous protocol with modification (36, 37), and then were submitted to Biotree Biotech Co., Ltd. (Shanghai, China) for detecting the metabolite concentrations by UPLC-Q-TOF/MS analysis. The bioinformatics analysis was performed according to the standard protocols of Biotree Biotech Co., Ltd. Details descriptions of the sample preparation, UPLC-Q-TOF/MS-based metabonomics, and data analysis are provided in the Supplementary Materials.

### Correlation Analysis Between Cecal Microbiota and Serum Metabolites

Relationships between different physiological indicators in the CHE hens were calculated by using Spearman correlations, and a Mantel test was employed to reveal their associations with cecal phyla. Those two analysis approaches were using a ggcor package (38) run in the R environment, and the figures were drawn using R package ggplot2 (39). Random forest algorithm was used to discriminate the serum metabolic profile of the samples from different groups based on the microbiota profile (genus-level-relative abundance data) using the R package “randomForest” with 1,000 trees and all default settings (40). The rfPermute function in the “rfPermute” R package was used to estimate the importance of these significant genera on the response variables (41, 42). Correlations between cecal genera among the three groups were calculated with the sparse correlations for compositional data algorithm (SparCC) implemented in a SparCC python module (43), and corresponding networks were plotted using the R package qgraph (44). All above analysis procedures are detailed in the Supplementary Materials.

## Results

### Physiological Alteration and Intestinal Injury in Hens Induced by CHE

Data from China Meteorological Administration (CMA)'s open dataset suggest that 25 major cities have experienced the maximum daytime temperature over 35°C in China from January to July 2018, among which 10 cities have suffered over 20 days of the highest temperature over 35°C (Figure 1A). This result showed animals in those areas have a greater risk of encounter chronic heat exposure. In the current study, no deaths or study discontinuations occurred in any of the experimental groups during a 21-day experimental period. We illustrated changes in serum biochemistry and antioxidant status of hens in response to heat exposure in Supplementary Table S2. According to the present results, there were no significant differences in levels of ALB and I-BIL among all groups at each time point. In addition, compared with the TN group, the levels of TP, HDL, T-AOC, SOD, and NO in group HS were significantly (*p* <0.05) decreased at all three points, whereas the levels of LDL, AST, and MDA were considerably (*p* < 0.05) increased. Similarly, the levels of D-BIL, TC, TG, CREA-S, UA, γ-GT, ALT, LDH, CK, and CK-MB were significantly (*p* < 0.05) higher, while the levels of ALP, GLO, HDL, and GSH-PX were significantly lower in the HS group for various periods of time (exposing Day 14 and Day 21) in hens relative to the TN group. Significant changes were not observed in all serum biochemistry, and antioxidant status in this study except in the level of TC and TG was significantly (*p* < 0.05) decreased in Group PF compared with Group TN on Day 14 and Day 21.

Next, as shown in Figure 1C, we detected remarkably high levels of HSP70 in serum of heat exposure chickens across all time points. In contrast, no significant differences between the groups TN and PF were found at each time point. A similar phenomenon was observed when detecting the protein expression levels of HSP70 in cecal tissues according to immunofluorescence staining (Figure 1D). Meanwhile, the histopathological report of the cecum of chickens is presented in Figure 1E. No obvious histopathological and oxidative injury was observed between TN and PF groups, but the morphology of cecum in the HS group showed a severe injury of intestinal, including shedding of the cecal mucosal epithelial cells into the intestinal cavity, shrinking the intestinal villi, and eventually damaging the arrangement of intestinal villi on Day21. All the above results suggested the HS chicken model was successful and could be used for further experiments.

### Altered Composition of the Gut Microbiota Induced by CHE in Hens

**Figure 2:**
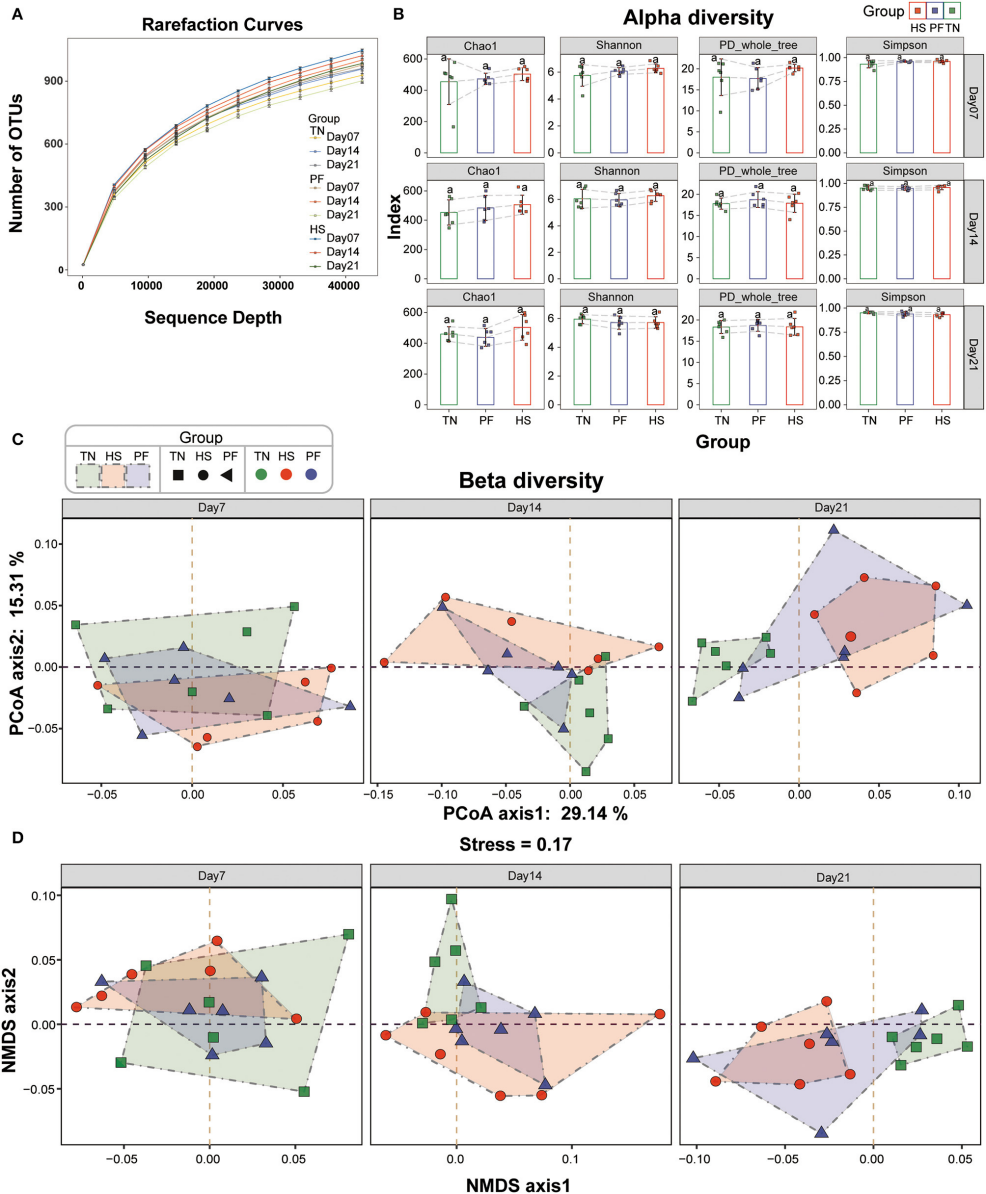
Comparisons of alpha diversity and beta diversity indices in cecal microbial communities of hens among different experimental groups. **(A)** Rarefaction curves of cecal bacteria from the three groups at each time point. **(B)** The α-diversity was compared among the TN, HS, and PF groups using Chao1, Shannon, PD_whole_tree, and Simpson diversity indices. Means with the same letter do not differ statistically; means with different letters are statistically different (*p* < 0.05). **(C)** A principal coordinate analysis (PCoA) plot of beta diversity based on the unweighted Unifrac distance matrices. **(D)** A non-metric multidimensional scaling (NMDS) plot of beta diversity based on the unweighted Unifrac distance matrices.

To characterize the effects of chronic heat exposure on the structure of the gut bacterial communities, the cecal microbiota of the hens was analyzed by sequencing the 16S rDNA variable region V4, which generated 4,059,602 sequences from 54 samples. After size filtering, quality control and chimera removal, we got 4,046,076 qualified sequences, with an average of 74,927 per sample and an average sequence length of approximate 252 bp (Supplementary Table S3). All remaining sequences were delineated into 1,137 operational taxonomic units (OTUs) at a similarity cutoff of 97%. Meanwhile, rarefaction curves of all samples reached saturation plateaus, suggesting that the current analysis had adequate depth to capture most microbial diversity information (Figure 2A).

Next, we calculated alpha diversity based on rarefied tables to analyze the complexity of species diversity in each sample by using several indices, including the Chao1, Shannon, PD_whole_tree, and Simpson indices. There was a trend toward a gradual increase of Chao1 index in the HS group compared to other groups across all time points, but the difference was not significant. Similarly, we did not observe any significant changes in other three indexes among the groups throughout the entire exposing period (Figure 2B). Beta diversity analysis was employed to evaluate the discrepancies of samples in species complexity by applying non-metric multidimensional scaling (NMDS), principal coordinate analysis (PCoA), and analysis of similarities (ANOSIM) based on the weighted UniFrac distance. The results from PCoA and NMDS plot revealed a clear separation of TN and HS hens on Day 21 (ANOSIM, *p* = 0.008, R = 0.7362). The same trend was also apparent on Day 7 (*p* = 0.556) and Day 14 (*p* = 0.147). But the difference between the two groups at those periods was not significant (Figure 2C; Supplementary Figure S2). Meanwhile, there was no clear separation in the clustering between the group PF and the other two groups at each time point (r = 0.104, *P* = 0.159). These results were further supported by our PCoA-based assessment of beta-diversity, which confirmed that chronic heat exposure altered the microbial community structure in a time-dependent fashion (Figure 2D; Supplementary Figure S2). Collectively, our results suggested that chronic heat exposure has barely affected alpha diversity of cecal microbiota in hens, but significantly altered the beta diversity over time, which was partly independent of decreased feed intake.

**Figure 3:**
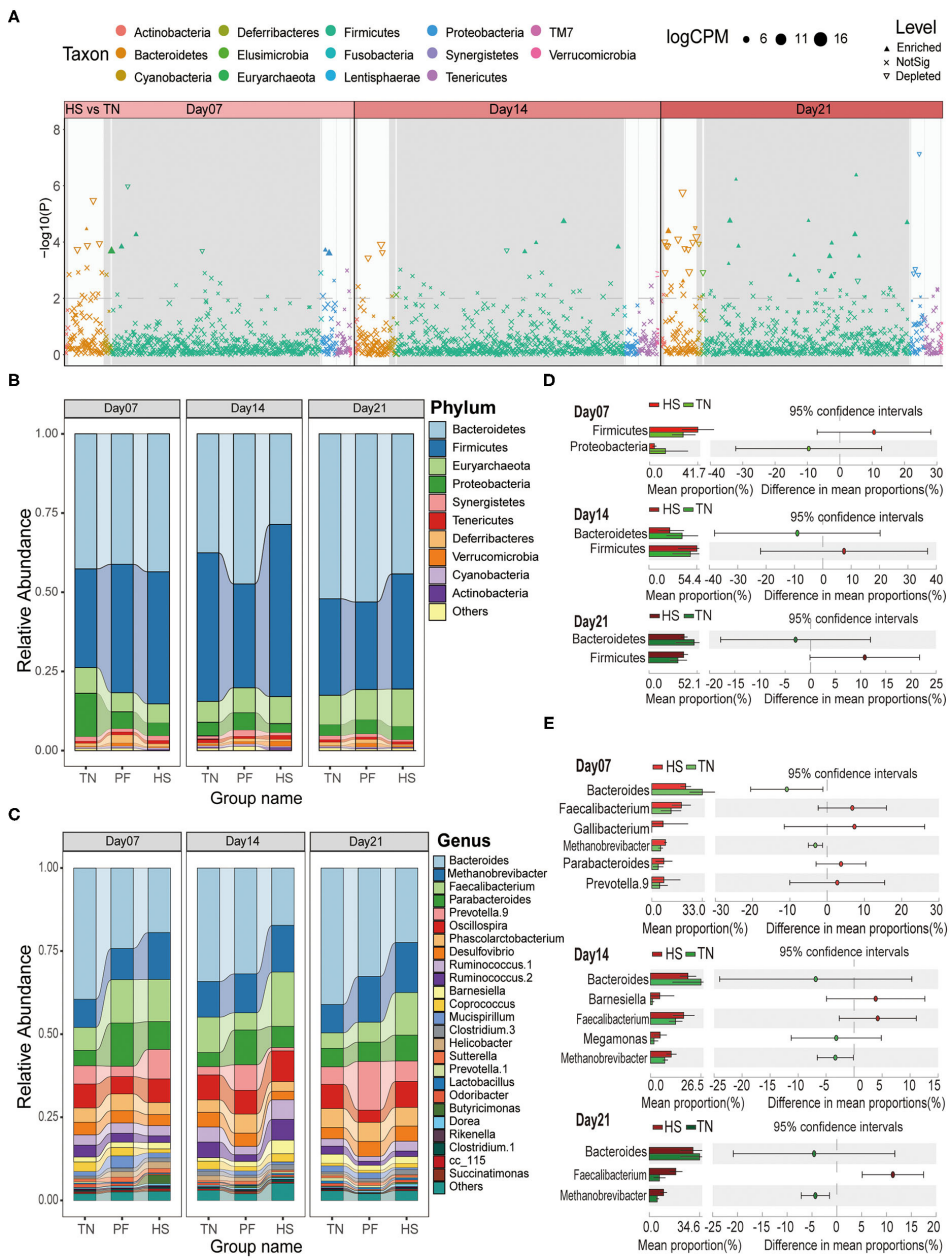
Effects of chronic heat exposure on cecal microbial composition at different taxonomic levels in growing hens. **(A)** The Manhattan plots show enrichment and depletion of microbial OTUs between HS and TN groups during the experimental period (on Days 7, 14, and 21). The dashed line represents the significant level (using false-discovery rate-corrected *P*-values of 0.01). The stacked bar plots represent the average distribution of bacterial phyla **(B)** and genera **(C)**, respectively. A statistical comparison between differences in the relative abundance of dominant microbial phyla **(D)** and genera **(E)** among three experimental groups based on Welch's *t*-test (*p* < 0.05 and effect sizes > 2).

To explain the observed differences in microbial clustering among groups, differential abundance analysis prepared using the edgeR package was used to compare the microbial taxa at the OTUs level. Compared with microbiota in the TN group, 39, 31, and 40 significantly enriched OTUs, and 30, 39, and 74 significantly depleted OTUs were detected in the HS group on Day 7, Day 14, and Day 21, respectively (*p* < 0.05, Supplementary Figures S3A–C). For samples in the PF group, compared with those in the TN group, it was enriched 40 and depleted 43 OTUs on Day 7, enriched 32 and depleted 48 OTUs on Day 14, enriched 21 and depleted 55 OTUs on Day 21, respectively (*p* < 0.05, Supplementary Figures S3D–E). Manhattan plots showed that these differential OTUs mainly belonged to four phyla, including *Firmicutes, Bacteroidetes, Proteobacteria*, and *Tenericutes* (*p* < 0.05, Figure 3A; Supplementary Figure S4A).

The taxonomic analysis at the phylum level showed that *Bacteroidetes* (40.42%) dominated the bacterial communities across all samples, followed by *Firmicutes* (40.19%), *Euryarchaeota* (8.27%), and *Proteobacteria* (4.81%) (Figure 3B). Then, we analyzed the potential difference in these phyla among three groups through Welch's *t*-test for STAMP analysis (Effect size > 2 and *p* < 0.05). The results revealed *Firmicutes* were enriched in the HS group through all time points as compared to the TN group, while *Bacteroidetes* were depleted after 14 and 21 days heat exposure (Figure 3C). For Group PF, *Firmicutes* were enriched on Day 7, but depleted on Day 14 and Day 21 compared to the TN group. No significant changes were observed about *Bacteroidetes* across all three time points except it depleted on day 14 (Supplementary Figure S4B). We also calculated the *Firmicutes*/*Bacteroidetes* (F/B) ratios among three groups at each time point. The result showed that, compared with the TN group, the F/B ratios consistently increased in the HS group, and exhibited a tendency to be decreased in the PF group (*p* < 0.05, Supplementary Figure S4D). Down to the genus level, we observed that *Bacteroides* (29.15%), *Methanobrevibacter* (11.76%), *Faecalibacterium* (9.80%), *Parabacteroides* (7.36%), and *Prevotella* 9 (6.09%) were the dominant genera in all the groups (Figure 3D). Further analysis showed that, compared with the TN group, the *Bacteroides* in Group HS have a lower abundant on each time point, while *Faecalibacterium* and *Methanobrevibacter* have a higher abundance throughout the experimental period (Welch's *t*-test, ES > 2 and *p* < 0.05, Figure 3E). Compared to the samples from the TN group, the *Prevotella.9* depleted in the PF group on Day 7 but enriched on Day 14 and Day 21 (Welch's *t*-test, ES > 2 and *p* < 0.05, Supplementary Figure S4C). All above results suggested chronic heat exposure can affect cecal microbiota composition of growing hens through changing the abundance of OTUs, which are mainly from phyla *Firmicutes* and *Bacteroidetes*.

**Figure 4:**
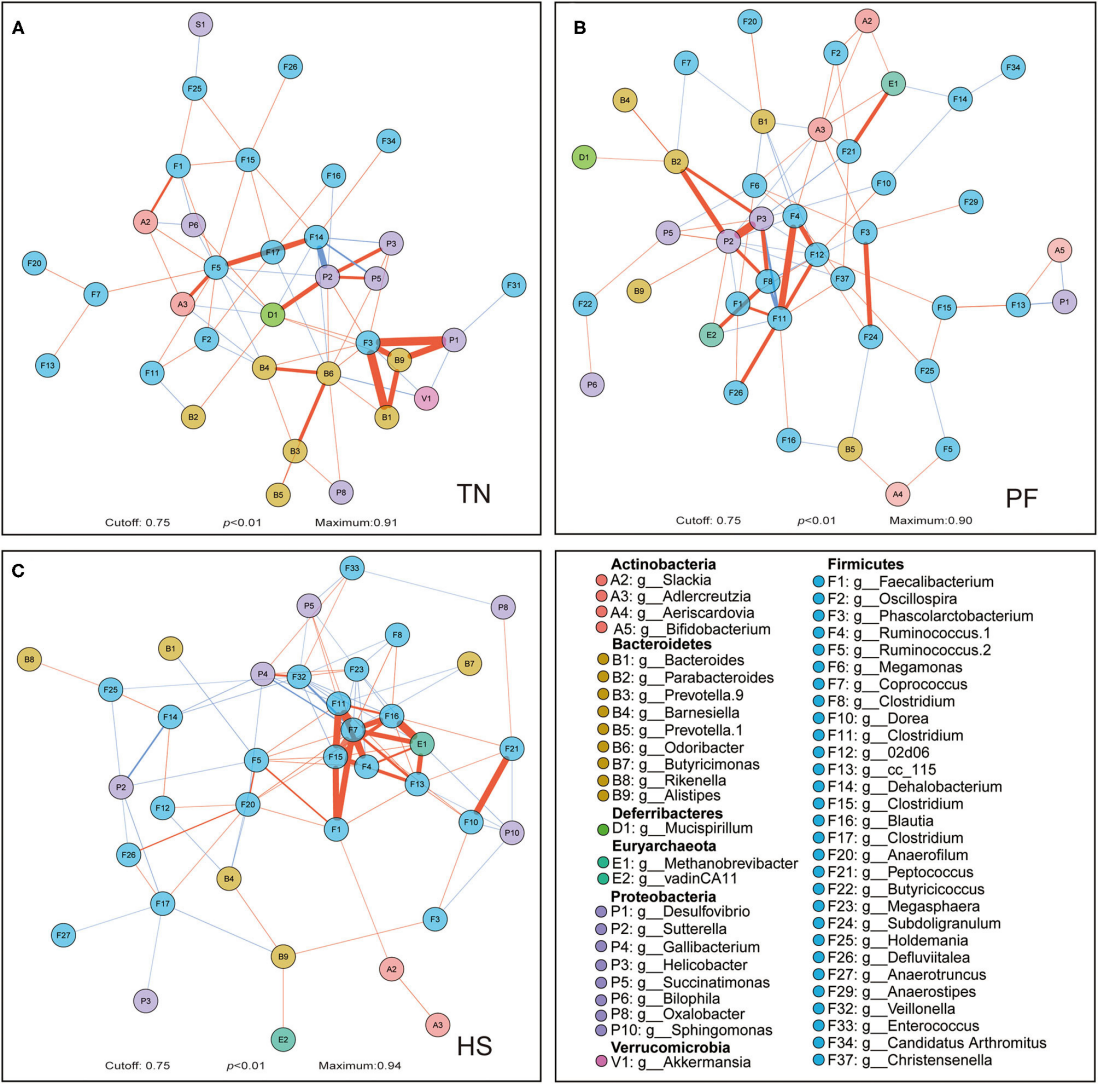
Correlation networks observed between genera. Correlations between microbial genera among TN **(A)**, PF **(B)**, and HS **(C)** groups were calculated with the Sparse Correlations for Compositional data algorithm (SparCC). The different colors of the nodes represent different phylum, which the genera belonging. The edge color represents positive (red) and negative (blue) correlations, and the width of the edge represents the magnitude of the correlation (wider if it is higher). Only high-confidence interactions with pseudo *p*-value ≤ 0.01 were drawn in the network using the R package qgraph.

We also constructed the genus-level microbial correlation network for each group based on SparCC (sparse correlations for compositional data) to reveal whether the chronic heat exposure changed the correlation and constructive interaction structures of gut microbiota. The network of HS and PF groups captured 37 nodes, 200 edges, 41 nodes, 158 edges, respectively, and has a slight difference from that in the TN group (34 nodes and 146 edges, Figure 4). We found that the inter-phyla correlation in HS and PF groups was weaker than that in the TN group (Figures 4A–C). For example, in the TN group hens, *Desulfovibrio* was positively correlated with *Alistipes*, but these correlations were not observed in the HS or PF group (Figures 4B,C). In the network of the HS group, several genera under phyla of *Firmicutes* have stronger positive correlation with each other, which showed heat exposure may weaken the inter-phyla interaction of genus that belongs to different phyla (Figure 4C). Altogether, these results suggest that the chronic heat exposure that significantly simplified microbial associations loosened the inter-phyla interacted relationships of cecal bacteria.

### Association Between Gut Microbial Phyla and Serum Physiologic Parameters

**Figure 5:**
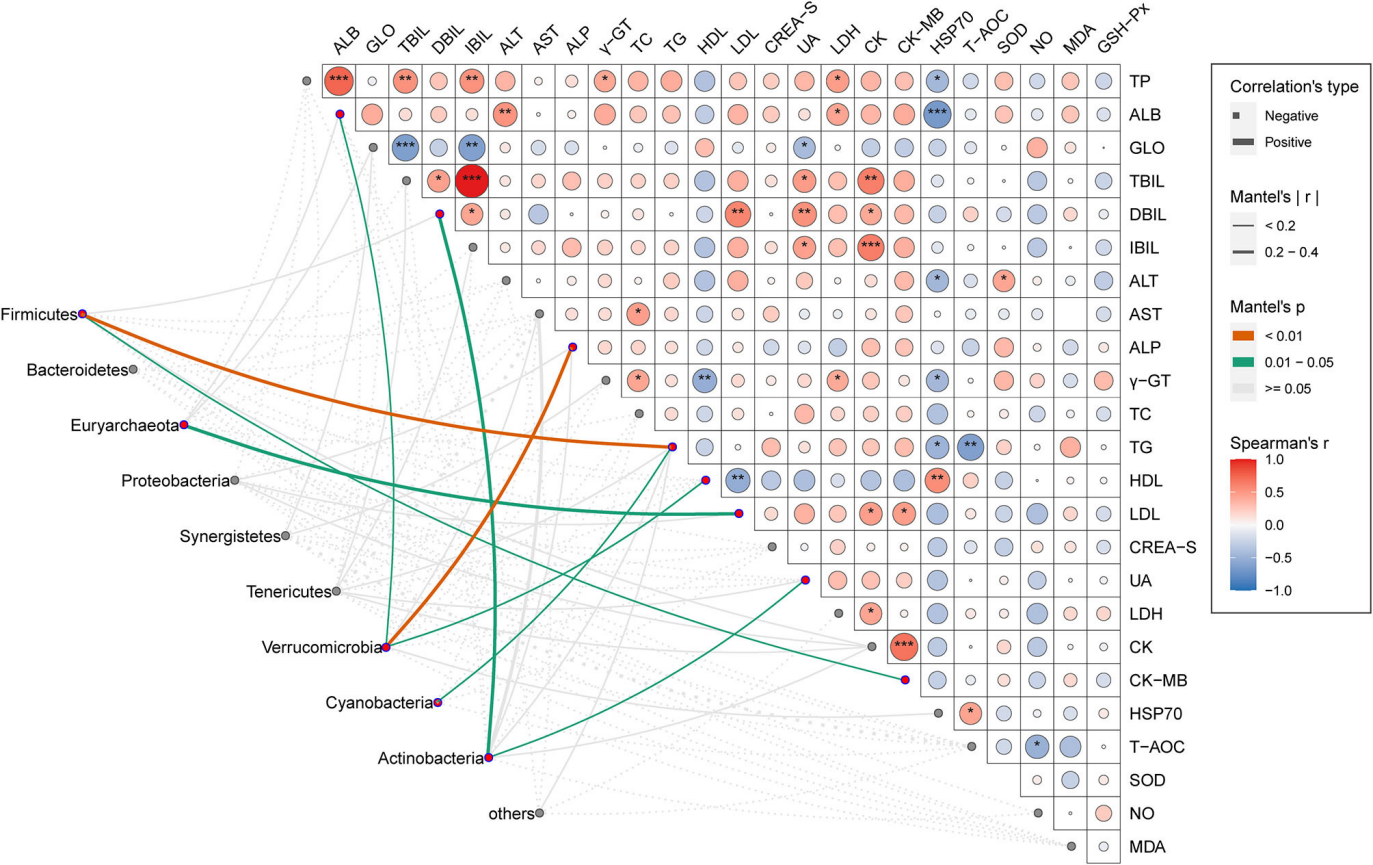
Association between cecal microbial phyla and serum physiologic parameters. Pair-wise comparisons of a serum physiologic parameter in CHE hens were shown with a color gradient denoting Spearman's correlation coefficients. The asterisk shows statistical significance. *p < 0.05; **p < 0.01; ***p < 0.001. Taxonomic groups (phyla) were linked to each serum physiologic parameter by using the Mantel test. The edge thickness denotes the Mantel's r statistic for the corresponding correlations, and the edge color represents the statistical significance (*P*-values) based on 999 permutations.

The mantel test was given to find out the interaction pattern between phylum-level dominant OTUs of gut microbiota and serum parameters of the host under chronic heat exposure (Figure 5). The results illustrated that the TG and ALP were significantly and positively influenced by *Firmicutes* and *Verrucomicrobia*, respectively (*p* < 0.01). As shown in Figure 5, the *Firmicutes* and *Verrucomicrobia* also had a significant effect on CK-MB, HDL, and ALB (*p* < 0.05), while we found no statistically meaningful links between these two phyla and the remaining serum parameters (*p* > 0.05). The *Euryarchaeota* and *Cyanobacteria* exhibited a strong positive interaction with LDL and TG (*p* < 0.05), respectively; *Actinobacteria* linked closely with UA and DBIL (*p* < 0.05). We observed no statistically significant associations between other microbial phyla (*Bacteroidetes, Proteobacteria, Synergistetes, Tenericutes*, and others) and serum parameters (*p* > 0.05). Through Pearson's correlation analysis, we explored the interconnections among serum parameters; results showed TG had a significant negative correlation with T-AOC and HSP70 (*p* < 0.01 or *p* < 0.05), while HDL was significantly and positively correlation with HSP70 but negatively related to LDL and γ-GT (*p* < 0.01). For LDL serum concentration, it showed significant positive relationships with DBIL, CK, and CK-MB (*p* < 0.01 or *p* < 0.05). We also observed several other significant associations, among which TC was significantly and positively linked to AST (*p* < 0.05); UA and CK had significant positive correlation with TBIL, DBIL, and IBIL (*p* < 0.001 or *p* < 0.01 or *p* < 0.05), while UA also showed a negative interaction with GLO. We observed the strongest correlation between the IBIL and TBIL, CK, and CK-MB, respectively. Overall, these results show that the interaction of microbial phyla and serum parameters is important in host lipid metabolisms.

### Serum Metabolic Profiles Changes Induced by CHE in Hens

The serum metabolite profiles were collected by electrospray ionization, applying both positive (POS) and negative (NEG) ion modes based on the UHPLC-QTOF-MS system, which can maximize the metabolite coverage and reduce background noise. The total ion chromatograms (TIC) of the serum samples in positive and negative ion modes both displayed stable retention times (Supplementary Figure S5). After peak alignment and removal of missing values, 1,761 and 1,337 valid peaks remained in POS and NEG scan modes, respectively. We then identified 440 (POS) and 276 (NEG) metabolites by matching these valid peaks with an in-house MS2 database.

**Figure 6:**
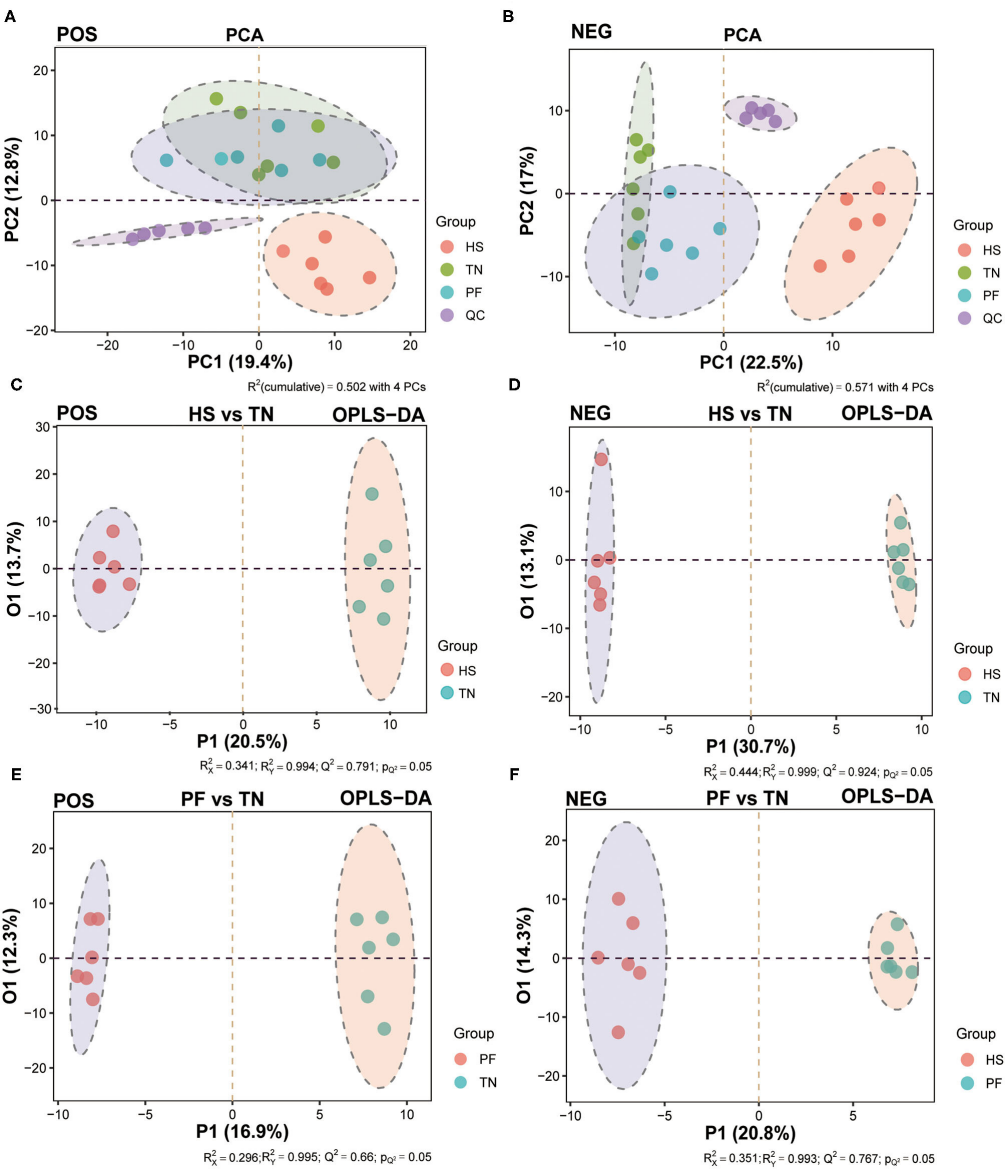
Multivariate analysis of the untargeted UHPLC-QTOF/MS metabolomics. The PCA score plots display the variance of positive **(A)** and negative **(B)** ions among TN, PF, and HS groups. The OPLS-DA score chart of serum metabolite analyzes the variance of positive **(C)** and negative **(D)** ions between the HS and TN groups, and the variance of positive **(E)** and negative **(F)** ions between the PF and TN groups.

The unsupervised principal component analysis (PCA) model was built under POS and NEG modes, respectively, to visualize similarities or latent differences among and within the groups. As shown in the PCA plots, a clear separation between the HS group and other groups was observed in the positive and negative modes (Figures 6A,B). As presented in the PCA score plots, in both POS and NEG modes, the samples from the chronic heat-exposed hens were readily separable from the rest, whereas the samples of PF hens also showed a separation trend with TN hens. Based on the above results, we further applied the OPLS-DA model to maximize the discrimination among three groups and develop a deeper understanding of the metabolites responsible for the separation. All samples in each score scatter plot of the PCA and OPLSDA model were within 95% confidence interval (a Hotelling's T-squared ellipse), which showed that the model can precisely describe the metabolic profiles of each group. The OPLS-DA scores plot also confirmed that each of the pair-wise groups (HS vs. TN, PF vs. TN, and HS vs. PF) was obviously separated in both positive and negative ion modes, showing that there were significant variations in the serum metabolic status of hens exposed to chronic high temperature (Figures 6C–F; Supplementary Figures S6A,B). The permutation test with 200 iterations showed that all R2 and Q2 values were smaller than the original points, which marked that all the OPLS-DA models in the present study were valid and the differential metabolites could be selected according to VIP value analysis (Supplementary Figures S6C–H).

**Figure 7:**
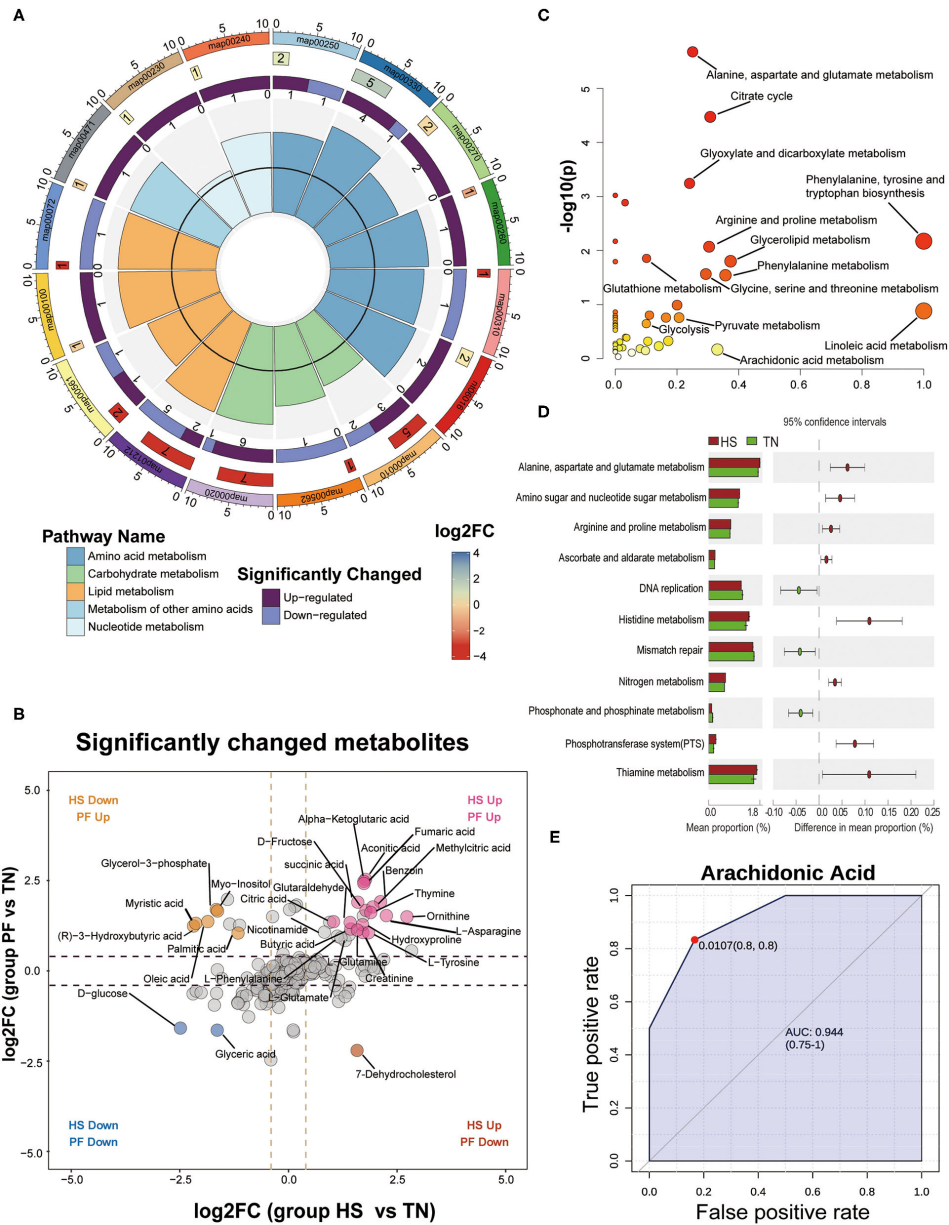
Detection and identification of differential metabolites and further classification into different metabolic pathways by KEGG analysis. **(A)** An enrichment cycle diagram (HS vs. TN) including four circles from the outside to the inside, representing a KO identifier, the number of differential metabolites in the categories, the number of upregulated and downregulated metabolites, and the max VIP value of differential metabolites in each category, respectively. **(B)** Scatterplot comparing the serum metabolites expression pattern between different groups (PF vs. TN at y-axis; HS vs. TN at x-axis). The significant changed metabolites were labeled in colors (|log 2-fold change| ≥ 1 and *p* < 0.01). **(C)** The bubble plots of significantly different metabolites enriched pathways between HS and TN groups. The abscissa and bubble size of the bubble represent the importance of the metabolic pathways in the topology analysis, and the ordinate and bubble color (from white to yellow to red) denote the significant level of the metabolic pathway through enrichment analysis. **(D)** Functional analysis of the cecal microbiota using PICRUSt (Level 3). The abundances of KEGG categories were significantly changed between HS and TN on Day 21. **(E)** A representative image of ROC curve analysis for differential metabolites in serum between HS and TN groups after 21-day heat exposure. More image of ROC curve analysis for differential metabolites among the three experimental groups is available in Supplementary Figure S7.

Referring to the principle combined with the variable importance of project (VIP) values > 1 and the two-tailed *p*-values calculated by Student's *T*-test < 0.05, we identified 40, 37, and 33 significant differential metabolites (SDMs) between each comparison pair-wise group (HS vs. TN, PF vs. TN, and HS vs. PF), respectively (Supplementary Table S4). Among which, compared with the TN group, 20 SDMs were upregulated and 2 SDMs were downregulated in both HS and PF groups, 1 SDM was upregulated in the HS group but downregulated in the PF group, 6 SDMs were downregulated in the HS group but upregulated in the PF group (Figure 7B). These results showed that the hens in the HS group and the PF group exhibited different metabolites accumulation patterns. The area under curve (AUC) index and receiver operating characteristic (ROC) analysis were further performed on the SDMs to seek potential biomarkers with AUC values of above 0.7. Finally, there were 14, 13, and 12 serum metabolites that were selected at the comparison pair-wise groups (HS vs. TN, PF vs. TN, and HS vs. PF), respectively. All the above potential biomarkers displayed high sensitivity and specificity, with AUC values range from 0.86 to 1.00 (Figure 7E; Supplementary Figures S7A–C).

Next, we performed a pathway enrichment analysis on the SDMs using MetaboAnalyst 4.0, which could describe the key pathways involved in hens in response to CHE. As shown in Figure 7C, 16 pathways were identified with pathway impact values over 0.10 and *p*-value < 0.05. Among which, 4 pathways belonged to lipid metabolism (fatty acid metabolism, glycerolipid metabolism, steroid biosynthesis, synthesis and degradation of ketone bodies), 6 pathways were part of amino acid metabolism (alanine, aspartate, and glutamate metabolism; arginine and proline metabolism; cysteine and methionine metabolism; glycine, serine, and threonine metabolism; lysine degradation; and phenylalanine and tyrosine metabolism), 3 pathways were part of carbohydrate metabolism (glycolysis, inositol phosphate metabolism, and the TCA cycle), 2 pathways belonged to nucleotide metabolism (purine metabolism and pyrimidine metabolism), and one pathway was part of metabolism of other amino acids (D-glutamine and D-glutamate metabolism) (Figure 7A). Those findings correspond with the PICRUSt2 that predicted the microbial function, suggesting cecal microbiota may affect a host serum metabolic profile by exerting physiological functions linked to amino acid metabolism (Figure 7D). Finally, a metabolic network of all SDMs gotten in serum samples was drawn manually to visualize the association between these metabolites (Supplementary Figure S8).

### Correlation Analysis of Microbial Genus Abundance With Serum Metabolites

**Figure 8:**
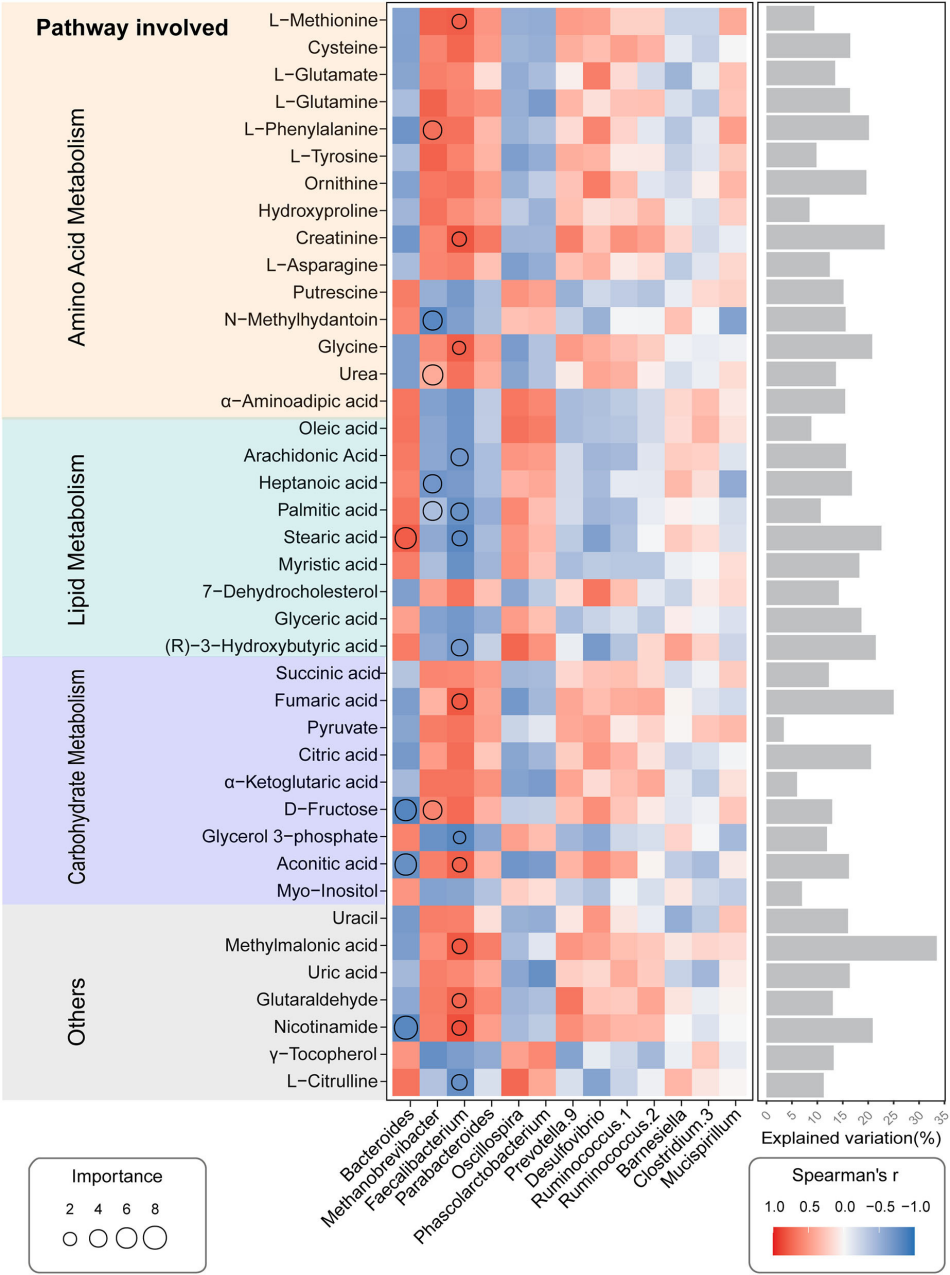
Potential biological contributions of the cecal microbial genera to a serum metabolic profile of the CHE hens based on correlation and the random forest model. Circle size represents the variable importance. Colors and sizes of squares represent R-value of Spearman's correlation. Bars in the right show the proportion of explained variability calculated via multiple regression modeling and variance decomposition analysis.

Using random forest regression modeling (RF) and spearman correlations analysis, we further checked the contributions and interaction pattern of the relative abundances of distinct genera in cecal microbiota on the variation of serum metabolics profiles. The relative abundance of different gut microbial genera can explain the changes in serum metabolite patterns (Figure 8). For instance, the abundance of *Faecalibacterium* genus, which belong to *Firmicutes* phyla, was the strongest predictor for amino acid metabolism-related (AA-related) metabolites in serum, including L-Methionine, Creatinine, and Glycine; Lipid Metabolism-related metabolites (LP-related), including Arachidonic Acid, Palmitic acid, Stearic acid, and (R)-3-Hydroxybutyric acid; Carbohydrate Metabolism-related metabolites (CH related), including Fumaric acid, Glycerol 3-phosphate, and Aconitic acid. Similarly, the *Methanobrevibacter* genera played a pivotal role in determining the concentration of several metabolites in serum, including L-Phenylalanine, N-methyihydantoin and Urea (AA related), Heptanoic acid, and Palmitic acid (LP related). The abundance of the *Bacteroides* was an important factor in predicting the serum level of metabolites, including Stearic acid (LP related), D-Fructose and Aconitic acid (CH related). Spearman correlation analysis showed that the abundance of *Faecalibacterium* and *Methanobrevibacter* genera exhibited a tendency to positive correlation with amino acid metabolism and carbohydrate metabolism-related metabolites but displayed a negative correlation with lipolysis-related metabolites. We observed an inverse trend in the abundance of the *Bacteroides* genera related to the serum metabolic profiles (Figure 8). These results suggest that distinct microbial genera abundance influenced the serum metabolites accumulation patterns. In particular, the F*aecalibacterium* and *Methanobrevibacter* were important variables for predicting serum metabolic profiles in hens exposure to high-ambient temperature.

## Discussion

Growing evidence showed chronic heat exposure impairs physiological processes of energy homeostasis in animals (45, 46), especially for avian species, resulting in lipid metabolic disorders, finally caused excessive fat accumulation (16, 47). The gut microbiota has been recognized as a risk pivotal factor contributing to obesity by affecting host energy harvest and storage (48, 49). Specially, the growing layer of hens is more sensitive to the high-temperature environment due to the immaturities in the anatomy and function of intestinal mucosa (50), which might be easily damaged by the heat exposure, resulting in unavoidable effect of the gastrointestinal microbiota (51, 52). Recent work by Cândido (53) has suggested that the temperature regulation systems in hens at the brooding phase (1–6 weeks of age) are not yet mature; high-humidity environment is requisite to avoid cold stress and improve chick survival. However, the thermoregulatory system becomes fully developed in growing-layer hens (7–17 weeks of age); thus, high-humidity environment changed to a prime risk factor to induce heat stress (53, 54). Although increasing studies have confirmed that high temperature is a contributor to the disorder of host metabolism and the gut microbiota disturbance, limited studies have investigated the interplay between hens and microbiota under high-ambient temperature (25, 55). To fill this gap of knowledge, the present study explored the potential interaction of the gut microbiota and serum metabolites in stressed-growing layer hens induced by the CHE using multi omics approaches.

The serum biochemical parameters respond to reflect the body to environmental stressors, and then they can be used as physiological biomarkers to know the situation of nutrient metabolism and organism health raised in stressed conditions (27, 56, 57). Here, we measured changes of some represented physiological biomarkers after 21 days of heat exposure in hens. Our results show that the lipid profiles (TG, TC, LDL, HDL) were significantly affected by the chronic heat exposure. This finding is consistent with previous studies, showing TG, TC, and LDL levels were significantly increased in chickens raised in CHE conditions; meanwhile, HDL was significantly decreased (27, 58). Lu et al. (20) stated that the excessive fat accumulation in birds under heat exposure is linked to chicken-breed-specific higher resistance to high-ambient temperature (20). Similar results were reported in mammals, where elevated ambient temperature can enhance lipid metabolism (59, 60). Another remarkable change observed in this study was the CHE can simultaneously affect multiple organ indexes (liver: ALT, AST, ALP, γ-GT, TBIL, DBIL, and IBIL; kidney: CREA-S and UA; heat: CK, LDH, and CK-MB), showing heat stress could cause dysfunction in multiple organs. These results concur with those of previous studies, suggesting response to CHE involves multiple organs and systems (23, 61). In the present study, we also examined molecular indices of oxidative stress; it showed that birds subjecting to chronic heat exposure increased oxidative stress, as shown by lower levels of T-AOC, SOD, and GSH-Px with higher MDA concentrations in serum. Paralleled with our findings, previous studies also reported that duration of thermal stress affects serum oxidative stress indices in a time-dependent manner (62). Lu et al. showed that CHE can cause damaged or malfunctioning mitochondria by generating reactive oxygen species (ROS), which may finally result in altered energy metabolism (47). HSP70 is considered as a reliable biological indicator of animal response to heat stress, which is the most abundant and temperature-sensitive heat shock protein (HSP) associated with heat exposure (63, 64). Consistent with previous studies, our results showed that not only the serum HSP70 level but also its expression in cecal tissue after CHE are significantly elevated, suggesting CHE success induced heat stress response in hens. Of note, compared with the TN group, no significant changes were observed in all of above physiological changes in the PF group, except for an inverse tendency observed in TG and TC, indicating that CHE-induced physiological function damage and metabolic disorders were partly independent from the reduced feed intake (65). Taken together, these results suggest the Candido HS model induced by CHE in hens was successfully established.

The avian gastrointestinal tract is populated by a diverse and complex microbial community (66), and the alterations in intestinal morphology and microenvironment are closely connected to the imbalance of gut microbiota (67, 68). Regarding the histopathological findings in the current work, severe damaging lesions were observed in the cecal tissues of hens after 21 days of heat exposure, and those intestinal morphology damages, meaning gut microbiome, might also have been affected by the CHE. Thus, we used 16S rRNA sequencing technology to determine whether the gut microbiota community in hens was affected by the CHE-induced heat stress. Our results show that the influence of heat stress was not manifested as changed alpha diversity of gut microbiome, but changed beta diversity in a time-dependent manner, showing that CHE could alter the structure and composition of cecal microbiota in hens (69). Going a step further, our result showed the two main dominant phyla altogether comprise above 80% of all the bacterial sequences at the end of the experiment, which agrees with previous studies of gut microbial profiles in birds (70, 71). The balance of *Firmicutes* and *Bacteroidetes* is measured as a ratio, *Firmicutes*/*Bacteroidetes* (F/B), which is a predictive marker reflecting gut microbiota dysbiosis (72, 73). Besides, the increment of the F/B ratio induced by the contraction of *Bacteroidetes* and/or expansion of *Firmicutes* is tightly associated with fat accumulation and potential for obesity both in humans and other animals (74, 75). Recently, several reports have suggested that the gut microbiota of stressed birds presented a significantly increased trend of the F/B ratio (76–78). In the current study, consistent with these previous reports, we observed an increase in *Firmicutes* and a decrease in *Bacteroidetes* after the CHE treatment at later two time points; meanwhile, the F/B ratio was incremented across all time points, showing the CHE can induce the gut microbiota dysbiosis and might further affect a lipid deposit in hens. However, a decrement of the F/B ratio was gotten in the PF group at each time point as compared to the TN group, which suggested that the changes of gut microbiota composition induced by the CHE are partly independent from the reduction of feed intake in hens. The result appears to contradict with Xing et al. (79), who noted the alterations in the gut flora of heat-stressed-laying hens are mainly linked with reduced feed intake (79). The discrepant chicken breeds, exposure time, and temperature of the study models may partly explain this divergence (20). These results have let us inferred the CHE can clearly induce dysbiosis of the gut microbial community reflected in the increment of the F/B ratio, which may consequently affect the lipid metabolism of hens.

According to the taxonomic analysis at the genus level, with increase of heat exposure time, the changing patterns of several genera were consistent across time points. Our results go further, showing that the relative abundance of *Bacteroides* decreased, whereas the relative abundance of *Faecalibacterium* and *Methanobrevibacter* increased after CHE treatment at all three time points. *Methanobrevibacter*, belongs to the phylum *Euryarchaeota*, is a common bacterial taxon inhabiting in the cecum of chickens (75), recognized as methane producing and energy-efficient genus (80). Wen et al. found that chickens with a higher *Methanobrevibacter* abundance had significantly higher abdominal fat content than those with a lower abundance of *Methanobrevibacter* (29). Recent original investigations have also highlighted the increment of genus *Methanobrevibacter* in heat-stressed animals might lead to excessive fat accumulation through enhanced energy capture of the host (75, 81). As for *Faecalibacterium* that belongs to the phylum *Firmicutes*, it was likely to be a biomarker for predicting the intestinal and systemic host health through its potential anti-inflammatory capability (82, 83). However, Niu et al. (84) reported that *Faecalibacterium* genus presents a positive relation with the abdominal fat and subcutaneous fat thickness in broiler chickens, and similar finding has been reported in the study of Gallardo-Becerra et al. (85), who observed *Faecalibacterium* was significantly increased in obesity and positively correlated to BMI in humans (85). Besides, Niu et al. (84) also reported that one taxon from the *Bacteroidetes*, genus *Bacteroides* related negatively with fat accumulation in broiler (84). Unsurprisingly, the altered patterns of genera in the PF group differed from the HS group as compared to the TN group, and it agrees to our results at the phylum level, which further confirmed that the gut microbiota dysbiosis caused by CHE treatment is partly independent from the reduced feed intake. Collectively, we hypothesized that the CHE treatment could alter the cecal microbial community composition by affecting the relative abundance of several specific genera, and it might further contribute to the lipid metabolism disturbance in hens.

To shed light on the results of biochemical level changes in serum of hens after 21 days of the CHE treatment, a sensitive untargeted approach for metabolomics study involved in the UHPLC-QTOF-MS method was used for global characterization of metabolic profiling of serum samples. The results showed that the serum level of D-glucose was considerably decreased in both HS and PF groups as compared to the TN group at the end of the experiment. Consistently, evidence has accumulated that heat stress can let various animal species into a state of negative energy balance (NEB), and this mainly associated with the reduced feed intake (86–89). Regarding this scenario, it results in the intense depletion of body energy reserves (90). However, Liao et al. (21) reported that the different breeds of cattle in the patterns of mobilized body reserves (body fat or muscle-derived proteins) to compensate for the heat stress-caused energy deficit may differ. In avian species, under the NEG state caused by HS, the energy output in broilers was increased by promoting the decomposition of muscle-derived proteins and mobilization of amino acids as fuel to meet their energy demands (12, 91). In parallel to these findings, we found that 10 metabolites were remarkably increased after the CHE treatment, which were mainly implicated in the amino acid metabolic process, including alanine, aspartate, and glutamate metabolism; arginine and proline metabolism; cysteine and methionine metabolism; glycine, serine, and threonine metabolism; phenylalanine and tyrosine metabolism; lysine degradation; and D-glutamine and D-glutamate metabolism (12). Among them, the concentration of eight metabolites was also increased in the PF group as compared to the TN group, such as creatinine, L-asparagine, Ornithine, Hydroxyproline, urea, L-tyrosine, L-phenylalanine, and L-glutamine. In addition, our results of the serum biochemical, which revealed the concentration of serum TG in hens was highest in the HS group among the three groups, could further support these findings. The similar results have been reported in the studies of heat-stressed pigs (92–94). Considering the above results, to meet the energy deficit caused by the CHE, we inferred the alterations of the serum metabolic profiles in CHE hens mainly involving the increased proteolysis rather than lipolysis, and this tendency could be a specific metabolic adaptation of the poultry.

The microorganisms inhabiting the intestine live in close contact rather than isolation with each other, and the complex interconnected microbial networks reflect the structure and functioning of gut microbial communities (95). Thus, the correlation network analysis was constructed to assess the structure of complex microbial communities and the potential interactions among microbial members. Our results revealed the inter-genus interaction pattern of gut microbiota in CHE hens differed from the other two groups, in conformity with previous observation that heat exposure can alter the potential interaction within the gut microbial communities of the host (96). Generally, the higher interaction network complexity can provide a more stable biological buffer to microbial communities against the changing environment (97). In the present study, the analysis of correlation network emphasized that there were sparser interconnections among genera belonging to different phylum in the hens chronically exposed to high temperature. This finding suggests that the gut microbial community in CHE hens might be more sensitive to the habitat environmental pressures. Conversely, we also found that the genera under phylum *Firmicutes* in CHE hens have more complex interactions with each other, and it probably attributed to the high-temperature exposure that substantially increased the abundance of phylum *Firmicutes* in the gut microbial community of hens. Previous studies revealed that the gut microbial community formed with a high F/B ratio and the interactions of specific relevant microbial members may contribute to obesity development and associated metabolic deteriorations of the host (98). Based on the above, we deduced the CHE treatment could simplify the inter-genus interactions among genera belonging to different phyla but complexify the interaction of genera within the phylum *Firmicutes*.

Emerging evidence suggests that alterations in the gut microbiome composition and functions are associated with physiological disorders of the host (99, 100). Mantel correlation analysis between gut microorganisms and physiological indicators in CHE hens showed that most bacterial phyla had a positive influence on host physiological processes. For example, we observed that the relative abundance of phylum *Firmicutes* was significantly and positively associated with serum TG. A recent study of Mahfuz et al. has shown the elevated level of serum TG involves a closely linked excessive fat accumulation in chickens (101). Moreover, the increased ratio of *Firmicutes*/*Bacteroidetes* has been reported to have a positive relation with obesity. In the present study, there was no significant correlation between the phylum *Bacteroidetes* and any of the physiological indicators, suggesting that the lipid metabolism disorder in our CHE hens might be attributed to the increase in abundance of phylum *Firmicutes* (102). Supportive of this, Armougom et al. demonstrated that specific enzymatic activities of obese individuals were found in the phylum *Firmicutes* rather than in *Bacteroidetes* (103). Additionally, LDL can inhibit the transportation of serum cholesterol by forming an oxidized ox-LDL, which is one of the primary risk factors causing atherosclerosis and coronary heart disease (104). Our results revealed that the phylum *Euryarchaeota* was significantly and positively associated with the serum level of LDL, suggesting it might also contribute to the excessive fat accumulation in CHE hens. Taken together, we concluded that the phylum *Firmicutes* and *Euryarchaeota* have a potential interaction with a serum lipid parameter, suggesting it might affect the lipid regulation and metabolism in the CHE hens.

Recent studies have suggested that the alterations in serum metabolome could mirror the discrepancies about the gut microbiome in animals under many disease progressions (105, 106). In our study, we were also curious about the interactions between the serum metabolites and cecal bacterial genera in CHE hens. Random forest analysis showed that both genus *Faecalibacterium* and *Methanobrevibacter* were important predictors of the CHE-induced lipid metabolism disorder. As aforementioned, *Faecalibacterium* belongs to *Firmicutes*, and it is a dominant member of gut microbiota, which might have a positive impact on fat synthesis and increased risk of excess lipid deposition in the chickens (82–84). We also observed that the hens under NEB condition caused by CHE, rather than initiated by lipolysis, increased the proteolysis to fill the energy deficit. Given this information, it is not surprising that the spearman correlations analysis showed *Faecalibacterium* negatively associated with lipolysis-related metabolites but positively associated with proteolysis metabolites in CHE hens. Our results also revealed a similar trend in the *Methanobrevibacter*, which agrees with the results of a previous study reporting that it positively related to abdominal fat content in chickens (75). In addition, the genus *Prevotella* might play a negative role in lipolysis, although the trends were not significant in metabolism of the CHE hens. This finding is consistent with a recent contribution, describing that *Prevotella* increases fat accumulation in pigs fed with formula diets (107). Putting together, we speculated that the *Faecalibacterium* and *Methanobrevibacte*r were the important contributors to the CHE-induced disorders of lipid metabolism in hens.

## Conclusion

In summary, we developed a CHE-induced model in growing layer hens to investigate the effect of prolonged exposure to high-ambient temperature on the gut microbiome and serum metabolome, as well as potential interactions between these two datasets. 16S rRNA gene sequencing analysis showed that the CHE can clearly induce dysbiosis of gut microbial community reflected in the increment of the F/B ratio, which may consequently affect the lipid metabolism of hens. Supportive of this observation, the serum metabolome analysis revealed the CHE caused an energy deficit in hens, and, to meet this gap, the alterations of the metabolites mainly involve the increased proteolysis rather than lipolysis, and this tendency could be a specific metabolic adaptation of the poultry. We also found that the above changes induced by CHE were partly independent from the reduction in feed intake. Mantel correlation analysis between gut microorganisms and physiological indicators showed that the phylum *Firmicutes* and *Euryarchaeota* have a potential interaction with a serum lipid parameter, suggesting it might affect the lipid regulation and metabolism in CHE hens. Random forest analysis showed that both genus *Faecalibacterium* and *Methanobrevibacter* were important predictors of the CHE-induced lipid metabolism disorder. However, there are several limitations in this study that need to be addressed in future pieces of research. First, because of the limited sample size, additional research using a larger hen sample size needs to be conducted to validate our findings. Second, the potential interaction observed between gut microbiome, host serum physiological indicators, and metabolic profiles needs further validation. Despite the limitations, our findings may contribute to a better understanding of the metabolic mechanisms underlying the energy metabolism imbalance caused by the CHE and provide novel insights into the host-microbes interactions and their effects on the metabolic adaptation of hens under chronic heat exposure.

## Data Availability Statement

The datasets presented in this study can be found in online repositories. The names of the repository/repositories and accession number(s) can be found in the article/Supplementary Material.

## Ethics Statement

The animal study was reviewed and approved by Animal Care and Use Committee of Jiangxi Agricultural University.

## Author Contributions

CZ: conceptualization, methodology, formal analysis, data curation, visualization, and writing—original draft. XGa: methodology, data curation, validation, and writing – review & editing. XC and GT: validation and formal analysis. CH: validation and visualization. LG and YZ: data curation, validation, and formal analysis. GH: resources and validation. PL: funding acquisition, project administration, resources, and supervision. XGu: funding acquisition, project administration, supervision, resources, and conceptualization. All authors contributed to the article and approved the submitted version.

## Funding

This work was supported by grants from the Key Programs of the Natural Science Foundation of Jiangxi Province of China (Grant No. 2017ACB20012) and the National Natural Science Foundation of China (Grant No. 31860723). Part of this work was also supported by the Technology System of Modern Agricultural Poultry Industry of Jiangxi Province (JXARS).

## Conflict of Interest

The authors declare that the research was conducted in the absence of any commercial or financial relationships that could be construed as a potential conflict of interest.

## Publisher's Note

All claims expressed in this article are solely those of the authors and do not necessarily represent those of their affiliated organizations, or those of the publisher, the editors and the reviewers. Any product that may be evaluated in this article, or claim that may be made by its manufacturer, is not guaranteed or endorsed by the publisher.

## References

[1] Johnson JS. Heat Stress Alters Animal Physiology and Post-absorptive Metabolism During Pre-and Postnatal Development. Kooser Dr Ames, IA: Iowa State University (2014).

[2] Delgado C. Rising demand for meat and milk in developing countries: implications for grasslands-based livestock production. In: McGilloway DA editor, Grassland: A Global Resource. Proceedings of the twentieth International Grassland Congress. Dublin: Wageningen Academic Publishers (2005). p. 29–39.

[3] Gerbens-Leenes P Nonhebel S Krol MS. Food consumption patterns and economic growth. Increasing affluence and the use of natural resources. Appetite. (2010) 55:597–608. 10.1016/j.appet.2010.09.013 20854862

[4] Buzingo D. Components of Energetic Efficiency Associated With Broiler and Layer Performance. Stillwater, OK: Oklahoma State University (2003).

[5] Zaboli G Huang X Feng X Ahn DU. How can heat stress affect chicken meat quality?–a review. Poult Sci. (2019) 98:1551–6. 10.3382/ps/pey399 30169735

[6] Sanchez-Casanova R Sarmiento-Franco L Segura-Correa J Phillips CJ. Effects of outdoor access and indoor stocking density on behaviour and stress in broilers in the subhumid tropics. Animals. (2019) 9:1016. 10.3390/ani9121016 31766675 PMC6940855

[7] Wolfenson D Bachrach D Maman M Graber Y Rozenboim I. Evaporative cooling of ventral regions of the skin in heat-stressed laying hens. Poult Sci. (2001) 80:958–64. 10.1093/ps/80.7.958 11469662

[8] Shehata AM Saadeldin IM Tukur HA Habashy WS. Modulation of heat-shock proteins mediates chicken cell survival against thermal stress. Animals. (2020) 10:2407. 10.3390/ani10122407 33339245 PMC7766623

[9] St-Pierre N Cobanov B Schnitkey G. Economic losses from heat stress by US livestock industries. J Dairy Sci. (2003) 86:E52–77. 10.3168/jds.S0022-0302(03)74040-533398462

[10] Key N Sneeringer S Marquardt D. Climate change, heat stress, and US dairy production. USDA-ERS Economic Research Report. (2014).28109597

[11] Nawab A Ibtisham F Li G Kieser B Wu J Liu W. Heat stress in poultry production: mitigation strategies to overcome the future challenges facing the global poultry industry. J Therm Biol. (2018) 78:131–9. 10.1016/j.jtherbio.2018.08.010 30509629

[12] Lu Z He X Ma B Zhang L Li J Jiang Y. Serum metabolomics study of nutrient metabolic variations in chronic heat-stressed broilers. Br J Nutr. (2018) 119:771–81. 10.1017/S0007114518000247 29569538

[13] He J Guo H Zheng W Xue Y Zhao R Yao W. Heat stress affects fecal microbial and metabolic alterations of primiparous sows during late gestation. J Anim Sci Biotechnol. (2019) 10:1–12. 10.1186/s40104-019-0391-0 31700622 PMC6827230

[14] He J Liu R Zheng W Guo H Yang Y Zhao R. High ambient temperature exposure during late gestation disrupts glycolipid metabolism and hepatic mitochondrial function tightly related to gut microbial dysbiosis in pregnant mice. Microb Biotechnol. (2021) 14:2116–29. 10.1111/1751-7915.13893 34272826 PMC8449678

[15] Baziz HA Geraert P Padilha J Guillaumin S. Chronic heat exposure enhances fat deposition and modifies muscle and fat partition in broiler carcasses. Poult Sci. (1996) 75:505–13. 10.3382/ps.0750505 8786940

[16] Geraert P Padilha J Guillaumin S. Metabolic and endocrine changes induced by chronic heatexposure in broiler chickens: growth performance, body composition and energy retention. Br J Nutr. (1996) 75:195–204. 10.1017/BJN19960124 8785198

[17] Renaudeau D Collin A Yahav S De Basilio V Gourdine J-L Collier R. Adaptation to hot climate and strategies to alleviate heat stress in livestock production. Animal. (2012) 6:707–28. 10.1017/S1751731111002448 22558920

[18] Temim S Chagneau A-M Peresson R Tesseraud S. Chronic heat exposure alters protein turnover of three different skeletal muscles in finishing broiler chickens fed 20 or 25% protein diets. J Nutr. (2000) 130:813–9. 10.1093/jn/130.4.813 10736335

[19] Jozefczuk S Klie S Catchpole G Szymanski J Cuadros-Inostroza A Steinhauser D. Metabolomic and transcriptomic stress response of Escherichia coli. Mol Syst Biol. (2010) 6:364. 10.1038/msb.2010.18 20461071 PMC2890322

[20] Lu Q Wen J Zhang H. Effect of chronic heat exposure on fat deposition and meat quality in two genetic types of chicken. Poult Sci. (2007) 86:1059–64. 10.1093/ps/86.6.1059 17495073

[21] Liao Y Hu R Wang Z Peng Q Dong X Zhang X. Metabolomics profiling of serum and urine in three beef cattle breeds revealed different levels of tolerance to heat stress. J Agric Food Chem. (2018) 66:6926–35. 10.1021/acs.jafc.8b01794 29905066

[22] Thursby E Juge N. Introduction to the human gut microbiota. Biochem J. (2017) 474:1823–36. 10.1042/BCJ20160510 28512250 PMC5433529

[23] Li Q Wan G Peng C Xu L Yu Y Li L. Effect of probiotic supplementation on growth performance, intestinal morphology, barrier integrity, and inflammatory response in broilers subjected to cyclic heat stress. Anim Sci J. (2020) 91:e13433. 10.1111/asj.13433 32671948

[24] Burkholder K Thompson K Einstein M Applegate T Patterson J. Influence of stressors on normal intestinal microbiota, intestinal morphology, and susceptibility to *Salmonella enteritidis* colonization in broilers. Poult Sci. (2008) 87:1734–41. 10.3382/ps.2008-00107 18753440

[25] He J He Y Pan D Cao J Sun Y Zeng X. Associations of gut microbiota with heat stress-induced changes of growth, fat deposition, intestinal morphology, and antioxidant capacity in ducks. Front Microbiol. (2019) 10:903. 10.3389/fmicb.2019.00903 31105682 PMC6498187

[26] He X Lu Z Ma B Zhang L Li J Jiang Y. Chronic heat stress damages small intestinal epithelium cells associated with the adenosine 5′-monophosphate-activated protein kinase pathway in broilers. J Agric Food Chem. (2018) 66:7301–9. 10.1021/acs.jafc.8b02145 29954175

[27] Mahmoud H Dawood M Assar M Ijiri D Ohtsuka A. Dietary Moringa oleifera improves growth performance, oxidative status, and immune related gene expression in broilers under normal and high temperature conditions. J Therm Biol. (2019) 82:157–63. 10.1016/j.jtherbio.2019.04.016 31128643

[28] Yi D Hou Y Tan L Liao M Xie J Wang L. N-acetylcysteine improves the growth performance and intestinal function in the heat-stressed broilers. Anim Feed Sci Technol. (2016) 220:83–92. 10.1016/j.anifeedsci.2016.07.014

[29] Wen C Yan W Sun C Ji C Zhou Q Zhang D. The gut microbiota is largely independent of host genetics in regulating fat deposition in chickens. ISME J. (2019) 13:1422–36. 10.1038/s41396-019-0367-2 30728470 PMC6775986

[30] Liu J-B Chen K Li Z-F Wang Z-Y Wang L. Glyphosate-induced gut microbiota dysbiosis facilitates male reproductive toxicity in rats. Sci Total Environ. (2022) 805:150368. 10.1016/j.scitotenv.2021.150368 34543792

[31] Rincel M Aubert P Chevalier J Grohard P-A Basso L De Oliveira CM. Multi-hit early life adversity affects gut microbiota, brain and behavior in a sex-dependent manner. Brain Behav Immun. (2019) 80:179–92. 10.1016/j.bbi.2019.03.006 30872090

[32] Wang M Xie Z Li L Chen Y Li Y Wang Y. Supplementation with compound polysaccharides contributes to the development and metabolic activity of young rat intestinal microbiota. Food Funct. (2019) 10:2658–75. 10.1039/C8FO02565G 31025991

[33] Xiao S-S Mi J-D Mei L Liang J Feng K-X Wu Y-B. Microbial diversity and community variation in the intestines of layer chickens. Animals. (2021) 11:840. 10.3390/ani11030840 33809729 PMC8002243

[34] Huang L Guo X Liu P Zhao Y Wu C Zhou C. Correlation between acute brain injury and brain metabonomics in dichlorvos-poisoned broilers. J Hazard Mater. (2022) 422:126849. 10.1016/j.jhazmat.2021.126849 34416688

[35] Cui M Qi C Yang L Zhang M Wang H She G. A pregnancy complication-dependent change in SIgA-targeted microbiota during third trimester. Food Funct. (2020) 11:1513–24. 10.1039/C9FO02919B 31994568

[36] Wang W Zhao L He Z Wu N Li Q Qiu X. Metabolomics-based evidence of the hypoglycemic effect of Ge-Gen-Jiao-Tai-Wan in type 2 diabetic rats via UHPLC-QTOF/MS analysis. J Ethnopharmacol. (2018) 219:299–318. 10.1016/j.jep.2018.03.026 29580854

[37] Guo L Kuang J Zhuang Y Jiang J Shi Y Huang C. Serum metabolomic profiling to revealed potential biomarkers for the diagnosis of fatty liver hemorrhagic syndrome in laying hens. Front Physiol. (2021) 12:3. 10.3389/fphys.2021.590638 33633583 PMC7900428

[38] Huang H Zhou L Chen J Wei T. ggcor: Extended Tools for Correlation Analysis and Visualization. Vienna: R Core Team (2020). p. 7.

[39] Wickham H Chang W Wickham MH. Package ‘Ggplot2'. Create Elegant Data Visualisations Using the Grammar of Graphics Version. Vienna: R Core Team. (2016). Vol. 2. p. 1–189.

[40] Rcolorbrewer S Liaw MA. Package ‘randomForest'. Berkeley, CA: University of California (2018).

[41] Archer E Archer ME. Package ‘rfPermute'. Vienna: R Core Team (2016).

[42] Jiao S Yang Y Xu Y Zhang J Lu Y. Balance between community assembly processes mediates species coexistence in agricultural soil microbiomes across eastern China. ISME J. (2020) 14:202–16. 10.1038/s41396-019-0522-9 31611655 PMC6908645

[43] Watts SC Ritchie SC Inouye M Holt KE. FastSpar: rapid and scalable correlation estimation for compositional data. Bioinformatics. (2019) 35:1064–6. 10.1093/bioinformatics/bty734 30169561 PMC6419895

[44] Epskamp S Cramer AO Waldorp LJ Schmittmann VD Borsboom D. qgraph: Network visualizations of relationships in psychometric data. J Stat Softw. (2012) 48:1–18. 10.18637/jss.v048.i04

[45] Rahimi J Mutua JY Notenbaert AM Marshall K Butterbach-Bahl K. Heat stress will detrimentally impact future livestock production in East Africa. Nature Food. (2021) 2:88–96. 10.1038/s43016-021-00226-8 37117410

[46] Gonzalez-Rivas PA Chauhan SS Ha M Fegan N Dunshea FR Warner RD. Effects of heat stress on animal physiology, metabolism, and meat quality: a review. Meat Sci. (2020) 162:108025. 10.1016/j.meatsci.2019.108025 31841730

[47] Lu Z He X Ma B Zhang L Li J Jiang Y. Chronic heat stress impairs the quality of breast-muscle meat in broilers by affecting redox status and energy-substance metabolism. J Agric Food Chem. (2017) 65:11251–8. 10.1021/acs.jafc.7b04428 29212325

[48] Bäckhed F Ding H Wang T Hooper LV Koh GY Nagy A. The gut microbiota as an environmental factor that regulates fat storage. Proc Nat Acad Sci. (2004) 101:15718–23. 10.1073/pnas.0407076101 15505215 PMC524219

[49] Wang Y Kuang Z Yu X Ruhn KA Kubo M Hooper LV. The intestinal microbiota regulates body composition through NFIL3 and the circadian clock. Science. (2017) 357:912–6. 10.1126/science.aan0677 28860383 PMC5702268

[50] Liu L Fu C Yan M Xie H Li S Yu Q. Resveratrol modulates intestinal morphology and HSP70/90, NF-κB and EGF expression in the jejunal mucosa of black-boned chickens on exposure to circular heat stress. Food Funct. (2016) 7:1329–38. 10.1039/C5FO01338K 26843443

[51] Wang S Mahfuz S Song H. Effects of flammulinavelutipes stem base on microflora and volatile fatty acids in caecum of growing layers under heat stress condition. Braz J Poult Sci. (2019) 21. 10.1590/1806-9061-2019-0989

[52] Zheng R Wang P Cao B Wu M Li X Wang H. Intestinal response characteristic and potential microbial dysbiosis in digestive tract of Bufo gargarizans after exposure to cadmium and lead, alone or combined. Chemosphere. (2021) 271:129511. 10.1016/j.chemosphere.2020.129511 33445016

[53] Cândido M. Thermal Tolerance, Performance, and Physiology of Egg-Type Pullets When Subjected to Different Levels of Heat Stress During the Brooding and Growing Phases Sequentially. Tese (Doutorado em Engenharia Agrícola) – Universidade Federal de Viçosa, Viçosa (2019).

[54] Li Q Yu Z Chen Z. Effect of heat stress on mitogen-activated protein kinases in the hypothalamic–pituitary–gonadal axis of developing Wenchang chicks. Poult Sci. (2020) 99:567–77. 10.3382/ps/pez499 32416843 PMC7587847

[55] Türk G Çeribaşi AO Simşek ÜG Çeribaşi S Güvenç M Kaya SÖ. Dietary rosemary oil alleviates heat stress-induced structural and functional damage through lipid peroxidation in the testes of growing Japanese quail. Anim Reprod Sci. (2016) 164:133–43. 10.1016/j.anireprosci.2015.11.021 26656503

[56] Bai X Dai S Li J Xiao S Wen A Hu H. Glutamine improves the growth performance, serum biochemical profile and antioxidant status in broilers under medium-term chronic heat stress. J Appl Poultry Res. (2019) 28:1248–54. 10.3382/japr/pfz091

[57] Guo WL Guo J-B Liu B-Y Lu JQ Chen M Liu B. Ganoderic acid A from Ganoderma lucidum ameliorates lipid metabolism and alters gut microbiota composition in hyperlipidemic mice fed a high-fat diet. Food Funct. (2020) 11:6818–33. 10.1039/D0FO00436G 32686808

[58] Attia Y Hassan R Tag El-Din A Abou-Shehema B. Effect of ascorbic acid or increasing metabolizable energy level with or without supplementation of some essential amino acids on productive and physiological traits of slow-growing chicks exposed to chronic heat stress. J Anim Physiol Anim Nutr. (2011) 95:744–55. 10.1111/j.1439-0396.2010.01104.x 21158953

[59] Wen X Wu W Fang W Tang S Xin H Xie J. Effects of long-term heat exposure on cholesterol metabolism and immune responses in growing pigs. Livest Sci. (2019) 230:103857. 10.1016/j.livsci.2019.103857

[60] He J Zheng W Tao C Guo H Xue Y Zhao R. Heat stress during late gestation disrupts maternal microbial transmission with altered offspring's gut microbial colonization and serum metabolites in a pig model. Environ Pollut. (2020) 266:115111. 10.1016/j.envpol.2020.115111 32663631

[61] Yan J Bao E Yu J. Heat shock protein 60 expression in heart, liver and kidney of broilers exposed to high temperature. Res Vet Sci. (2009) 86:533–8. 10.1016/j.rvsc.2008.09.002 18951595

[62] Wang J Xue X Liu Q Zhang S Peng M Zhou J. Effects of duration of thermal stress on growth performance, serum oxidative stress indices, the expression and localization of ABCG2 and mitochondria ROS production of skeletal muscle, small intestine and immune organs in broilers. J Therm Biol. (2019) 85:102420. 10.1016/j.jtherbio.2019.102420 31657761

[63] Maak S Melesse A Schmidt R Schneider F Von Lengerken G. Effect of long-term heat exposure on peripheral concentrations of heat shock protein 70 (Hsp70) and hormones in laying hens with different genotypes. Br Poult Sci. (2003) 44:133–8. 10.1080/0007166031000085319 12737235

[64] Gu X Hao Y Wang X. Overexpression of heat shock protein 70 and its relationship to intestine under acute heat stress in broilers: 2. Intestinal oxidative stress. Poult Sci. (2012) 91:790–9. 10.3382/ps.2011-01628 22399716

[65] Xiong Y Yi H Wu Q Jiang Z Wang L. Effects of acute heat stress on intestinal microbiota in grow-finishing pigs, and associations with feed intake and serum profile. J Appl Microbiol. (2020) 128:840–52. 10.1111/jam.14504 31671233

[66] Pandit RJ Hinsu AT Patel NV Koringa PG Jakhesara SJ Thakkar JR. Microbial diversity and community composition of caecal microbiota in commercial and indigenous Indian chickens determined using 16s rDNA amplicon sequencing. Microbiome. (2018) 6:1–13. 10.1186/s40168-018-0501-9 29935540 PMC6015460

[67] Abu-Ali GS Mehta RS Lloyd-Price J Mallick H Branck T Ivey KL. Metatranscriptome of human faecal microbial communities in a cohort of adult men. Nat Microbiol. (2018) 3:356–66. 10.1038/s41564-017-0084-4 29335555 PMC6557121

[68] Giannenas I Bonos E Skoufos I Tzora A Stylianaki I Lazari D. Effect of herbal feed additives on performance parameters, intestinal microbiota, intestinal morphology and meat lipid oxidation of broiler chickens. Br Poult Sci. (2018) 59:545–53. 10.1080/00071668.2018.1483577 29873243

[69] Hu Y Huang X Zong X Bi Z Cheng Y Xiao X. Chicory fibre improves reproductive performance of pregnant rats involving in altering intestinal microbiota composition. Appl Microbiol. (2020) 129:1693–705. 10.1111/jam.14679 32356327

[70] Zhou C Xu P Huang C Liu G Chen S Hu G. Effects of subchronic exposure of mercuric chloride on intestinal histology and microbiota in the cecum of chicken. Ecotoxicol Environ Saf. (2020) 188:109920. 10.1016/j.ecoenv.2019.109920 31733937

[71] Huang C Shi Y Zhou C Guo L Liu G Zhuang Y. Effects of subchronic copper poisoning on cecal histology and its microflora in chickens. Front Microbiol. (2021) 12:739577. 10.3389/fmicb.2021.739577 34566941 PMC8456085

[72] Eckburg PB Bik EM Bernstein CN Purdom E Dethlefsen L Sargent M. Diversity of the human intestinal microbial flora. Science. (2005) 308:1635–8. 10.1126/science.1110591 15831718 PMC1395357

[73] Liu J Dong C Zhai Z Tang L Wang L. Glyphosate-induced lipid metabolism disorder contributes to hepatotoxicity in juvenile common carp. Environ Pollut. (2021) 269:116186. 10.1016/j.envpol.2020.116186 33302084

[74] Ley RE Turnbaugh PJ Klein S Gordon JI. Human gut microbes associated with obesity. Nature. (2006) 444:1022–3. 10.1038/4441022a 17183309

[75] Xiang H Gan J Zeng D Li J Yu H Zhao H. Specific microbial taxa and functional capacity contribute to chicken abdominal fat deposition. Front Microbiol. (2021) 12:569. 10.3389/fmicb.2021.643025 33815329 PMC8010200

[76] Wang X Feng J Zhang M Li X Ma D Chang SS. Effects of high ambient temperature on the community structure and composition of ileal microbiome of broilers. Polut Sci. (2018) 97:2153–8. 10.3382/ps/pey032 29562351

[77] Shi D Bai L Qu Q Zhou S Yang M Guo S. Impact of gut microbiota structure in heat-stressed broilers. Poult Sci. (2019) 98:2405–13. 10.3382/ps/pez026 30715508

[78] Liu G Zhu H Ma T Yan Z Zhang Y Geng Y. Effect of chronic cyclic heat stress on the intestinal morphology, oxidative status and cecal bacterial communities in broilers. J Therm Biol. (2020) 91:102619. 10.1016/j.jtherbio.2020.102619 32716869

[79] Xing S Wang X Diao H Zhang M Zhou Y Feng J. Changes in the cecal microbiota of laying hens during heat stress is mainly associated with reduced feed intake. Poult Sci. (2019) 98:5257–64. 10.3382/ps/pez440 31399742

[80] Michels N Van De Wiele T Fouhy F O'mahony S Clarke G Keane J. Gut microbiome patterns depending on children's psychosocial stress: Reports versus biomarkers. Brain Behav Immun. (2019) 80:751–62. 10.1016/j.bbi.2019.05.024 31112792

[81] Sales GFC Carvalho BF Schwan RF De Figueiredo Vilela L Meneses JM Gionbelli MP. Heat stress influence the microbiota and organic acids concentration in beef cattle rumen. J Therm Biol. (2021) 97:102897. 10.1016/j.jtherbio.2021.102897 33863450

[82] Angelakis E Armougom F Million M Raoult DJFM. The relationship between gut microbiota and weight gain in humans. Future Microbiol. (2012) 7:91–109. 10.2217/fmb.11.142 22191449

[83] Wu Y Lei Z Wang Y Yin D Aggrey SE Guo Y. Metabolome and microbiota analysis reveals the conducive effect of *Pediococcus acidilactici* BCC-1 and Xylan Oligosaccharides on Broiler Chickens. Front Microbiol. (2021) 12:1337. 10.3389/fmicb.2021.683905 34122394 PMC8192963

[84] Niu J Zhang J Wei L Ma X Zhang W Nie C. Cottonseed meal fermented by Candida tropical reduces the fat deposition in white-feather broilers through cecum bacteria-host metabolic cross-talk. Appl Microbiol Biotechnol. (2020) 104:4345–57. 10.1007/s00253-020-10538-7 32232527

[85] Gallardo-Becerra L Cornejo-Granados F García-López R Valdez-Lara A Bikel S Canizales-Quinteros S. Metatranscriptomic analysis to define the Secrebiome, and 16S rRNA profiling of the gut microbiome in obesity and metabolic syndrome of Mexican children. Microb Cell Fact. (2020) 19:1–18. 10.1186/s12934-020-01319-y 32143621 PMC7060530

[86] Ronchi B Bernabucci U Lacetera NG Nardone A. Effects of heat stress on metabolic-nutritional status of Holstein cows. Zootecnica e Nutrizione Animale. (1997) 13:128–32.

[87] Kikusato M Maekawa T Shirakawa H Toyomizu M Md. Abul Kalam A. Metabolic characteristics and oxidative damage to skeletal muscle in broiler chickens exposed to chronic heat stress. Comp Biochem Physiol A Mol Integr Physiol. (2009) 155:401–6. 10.1016/j.cbpa.2009.12.011 20036750

[88] O'brien M Rhoads R Sanders S Duff G Baumgard L. Metabolic adaptations to heat stress in growing cattle. Domest Anim Endocrinol. (2010) 38, 86–94. 10.1016/j.domaniend.2009.08.005 19783118

[89] Sabés-Alsina M Tallo-Parra O Mogas MT Morrell JM Lopez-Bejar M. Heat stress has an effect on motility and metabolic activity of rabbit spermatozoa. Anim Reprod Sci. (2016) 173:18–23. 10.1016/j.anireprosci.2016.08.004 27530369

[90] Ibrahim M Ayoub D Wasselin T Van Dorsselaer A Le Maho Y Raclot T. Alterations in rat adipose tissue transcriptome and proteome in response to prolonged fasting. Biol Chem. (2020) 401:389–405. 10.1515/hsz-2019-0184 31398141

[91] Zuo J Xu M Abdullahi YA Ma L Zhang Z Feng D. Constant heat stress reduces skeletal muscle protein deposition in broilers. J Sci Food Agric. (2015) 95:429–36. 10.1002/jsfa.6749 24871527

[92] Zhao L Mcmillan RP Xie G Giridhar SG Baumgard LH El-Kadi S. Heat stress decreases metabolic flexibility in skeletal muscle of growing pigs. Am J Physiol Regulat Integr Compar Physiol. (2018) 315:R1096–106. 10.1152/ajpregu.00404.2017 30256682

[93] Cui Y Wang C Hao Y Gu X Wang H. Chronic heat stress induces acute phase responses and serum metabolome changes in finishing pigs. Animals. (2019) 9:395. 10.3390/ani9070395 31261803 PMC6680871

[94] Fang W. Effects of Dietary Fiber and Heat Stress on Cholesterol and Bile Acids Metabolism in Pigs. Liège, Belgique: Université de Liège (2019).

[95] Tremaroli V Bäckhed F. Functional interactions between the gut microbiota and host metabolism. Nature. (2012) 489:242–9. 10.1038/nature11552 22972297

[96] Cao Y Liu Y Dong Q Wang T Niu C. Alterations in the gut microbiome and metabolic profile in rats acclimated to high environmental temperature. Microb Biotechnol. (2021) 15:276–88. 10.1111/1751-7915.13772 33620148 PMC8719808

[97] Ridlon JM Alves JM Hylemon PB Bajaj JS. Cirrhosis, bile acids and gut microbiota: unraveling a complex relationship. Gut Microbes. (2013) 4:382–7. 10.4161/gmic.25723 23851335 PMC3839982

[98] Zhang C Yin A Li H Wang R Wu G Shen J. Dietary modulation of gut microbiota contributes to alleviation of both genetic and simple obesity in children. EBioMedicine. (2015) 2:968–84. 10.1016/j.ebiom.2015.07.007 26425705 PMC4563136

[99] Lv X-C Guo W-L Li L Yu X-D Liu B. Polysaccharide peptides from Ganoderma lucidum ameliorate lipid metabolic disorders and gut microbiota dysbiosis in high-fat diet-fed rats. J Funct Foods. (2019) 57:48–58. 10.1016/j.jff.2019.03.043

[100] Agus A Clément K Sokol H. Gut microbiota-derived metabolites as central regulators in metabolic disorders. Gut. (2021) 70:1174–82. 10.1136/gutjnl-2020-323071 33272977 PMC8108286

[101] Mahfuz S He T Liu S Wu D Long S Piao X. Dietary inclusion of mushroom (*Flammulina velutipes*) stem waste on growth performance, antibody response, immune status, and serum cholesterol in broiler chickens. Animals. (2019) 9:692. 10.3390/ani9090692 31533253 PMC6770792

[102] Li J Wu T Li N Wang X Chen G Lyu X. Bilberry anthocyanin extract promotes intestinal barrier function and inhibits digestive enzyme activity by regulating the gut microbiota in aging rats. Food Funct. (2019) 10:333–43. 10.1039/C8FO01962B 30575836

[103] Armougom F Henry M Vialettes B Raccah D Raoult D. Monitoring bacterial community of human gut microbiota reveals an increase in Lactobacillus in obese patients and Methanogens in anorexic patients. PLoS ONE. (2009) 4:e7125. 10.1371/journal.pone.0007125 19774074 PMC2742902

[104] Niu J-L Zhang J Wei L-Q Zhang W-J Nie C-X. Effect of fermented cottonseed meal on the lipid-related indices and serum metabolic profiles in broiler chickens. Animals. (2019) 9:930. 10.3390/ani9110930 31703286 PMC6912724

[105] Liu R Hong J Xu X Feng Q Zhang D Gu Y. Gut microbiome and serum metabolome alterations in obesity and after weight-loss intervention. Nat Med. (2017) 23:859–68. 10.1038/nm.4358 28628112

[106] Clos-Garcia M Andrés-Marin N Fernández-Eulate G Abecia L Lavín JL Van Liempd S. Gut microbiome and serum metabolome analyses identify molecular biomarkers and altered glutamate metabolism in fibromyalgia. EBioMedicine. (2019) 46:499–511. 10.1016/j.ebiom.2019.07.031 31327695 PMC6710987

[107] Chen C Fang S Wei H He M Fu H Xiong X. Prevotella copri increases fat accumulation in pigs fed with formula diets. Microbiome. (2021) 9:1–21. 10.1186/s40168-021-01110-0 34419147 PMC8380364

