# Supplementary material for: Gut Microbiota and Serum Metabolite Potential Interactions in Growing Layer Hens Exposed to High-Ambient Temperature
Source: Front Nutr. 2022 Apr 27;9:877975. doi: 10.3389/fnut.2022.877975 (PMC9093710; doi:10.3389/fnut.2022.877975)
Supplement: Supplementary file 1 [file Table_1.docx]

**Table S1. Composition and Calculated Analysis of the Layer Basal Diet**

| **Ingredient** | **Percentage (%)** |
| --- | --- |
| Corn | 44.85 |
| Soybean meal, 48% CP | 34.52 |
| Meat and bone meal | 2.49 |
| DDGS | 1.99 |
| Soy oil | 4.40 |
| DL-Methionine | 0.27 |
| Limestone, small | 4.41 |
| Limestone, large | 4.41 |
| Dicalcium phosphate | 1.18 |
| Salt | 0.42 |
| Vitamin and mineral premix^1^ | 0.75 |
| Titanium dioxide | 0.30 |
| **Calculated composition** | **Percentage (%)** |
| Metabolizable energy (kcal/kg) | 2900 |
| Crude protein | 22.40 |
| Calcium | 4.00 |
| Non-phytate phosphorus | 0.45 |
| Fat | 6.85 |
| Digestible Met + Cys | 0.88 |
| Digestible lysine | 1.11 |
| Digestible threonine | 0.77 |
| **Analyzed composition** | **Percentage (%)** |
| Crude protein | 22.50 |
| Fat | 3.63 |

^1^Provided per kg of diet: vitamin A, 6595.69 IU; vitamin D3, 2209.56 ICU; vitamin E, 1.65 IU; vitamin B12, 6.60 μg; menadione, 1.15 mg; riboflavin, 4.12 mg; D-pantotheic acid, 6.07 mg; niacin,19.79 mg; choline, 381.68 mg; Co, 0.25 mg; Cu, 4.04 mg; I, 1.00 mg; Fe, 50.65 mg; Mn, 64.26 mg; Zn,48.69 mg.

| **Table S2 Effects of chronic heat stress on serum biochemical and anti-oxidative parameters of laying hens.** | | | | | | | | | |
| --- | --- | --- | --- | --- | --- | --- | --- | --- | --- |
| Parameters | Day7 | | | Day14 | | | Day21 | | |
|  | TN | HS | PF | TN | HS | PF | TN | HS | PF |
| TP (g/L) | 50.88±3.76^b^ | 41.71±2.41^a^ | 49.19±1.54^b^ | 56.23±3.95^b^ | 44.32±2.66^a^ | 52.06±6.55^b^ | 65.38±2.57^b^ | 52.21±4.61^a^ | 62.85±3.68^b^ |
| ALB (g/L) | 13.68±1.22^a^ | 14.00±0.73^a^ | 14.19±0.6^a^ | 15.04±0.54^a^ | 15.00±1.32^a^ | 14.42±0.97^a^ | 15.72±1.10^a^ | 16.19±0.87^a^ | 15.77±1.61^a^ |
| GLO(μmol/L) | 33.97±2.94^a^ | 30.32±3.86^a^ | 33.76±3.70^a^ | 37.28±2.18^b^ | 31.82±6.09^a^ | 36.06±2.48^ab^ | 40.59±5.66^b^ | 29.47±3.84^a^ | 35.03±3.40^ab^ |
| TC (mmol/L) | 2.83±0.23^a^ | 3.07±0.30^a^ | 2.88±0.25^a^ | 2.91±0.33^a^ | 3.44±0.31^b^ | 2.38±0.35^c^ | 3.04±0.30^a^ | 3.53±0.37^b^ | 1.93±0.27^c^ |
| TG (mmol/L) | 4.99±0.16^a^ | 5.07±0.32^a^ | 4.72±0.14^a^ | 5.05±0.17^a^ | 5.52±0.32^b^ | 3.49±0.08^c^ | 5.16±0.38^a^ | 5.93±0.54^b^ | 3.11±0.21^c^ |
| HDL(mmol/L) | 1.89±0.21^b^ | 1.68±0.08^a^ | 1.84±.12^ab^ | 1.80±0.20^b^ | 1.47±0.24^a^ | 1.72±0.13^ab^ | 1.74±0.20^b^ | 1.37±0.15^a^ | 1.67±0.12^b^ |
| LDL(mmol/L) | 1.56±0.09^a^ | 1.77±0.17^b^ | 1.45±0.16^a^ | 1.64±0.13^b^ | 1.94±0.08^c^ | 1.28±0.13^a^ | 1.65±0.07^b^ | 2.11±0.30^c^ | 1.10±0.09^a^ |
| ALT (U/L) | 4.11±0.52^a^ | 5.22±1.51^a^ | 4.49±1.26^a^ | 4.03±0.64^a^ | 5.67±1.38^b^ | 4.43±0.42^a^ | 4.25±0.67^a^ | 6.70±1.02^b^ | 4.67±0.79^a^ |
| AST (U/L) | 180.47±14.53^a^ | 212.76±26.39^b^ | 185.26±12.44^a^ | 174.12±13.39^a^ | 217.41±50.37^b^ | 192.33±15.46^ab^ | 178.53±23.50^a^ | 218.04±25.06^b^ | 188.25±26.82^ab^ |
| ALP(U/L) | 925.34±86.44^a^ | 804.07±93.99^a^ | 823.49±110.24^a^ | 982.27±68.26^b^ | 812.21±112.23^a^ | 883.94±86.17^ab^ | 1098.55±126.74^b^ | 791.42±123.41^a^ | 839.42±73.41^a^ |
| γ-GT (U/L) | 17.83±2.39^a^ | 20.30±1.87^a^ | 17.64±2.01^a^ | 19.51±1.70^a^ | 23.61±2.84^b^ | 19.40±1.34^a^ | 19.22±3.05^a^ | 24.85±3.55^b^ | 20.64±2.50^a^ |
| TBIL(μmol/L) | 24.98±3.52^a^ | 29.34±3.86^a^ | 25.27±2.64^a^ | 26.73±4.49^a^ | 32.90±5.00^a^ | 26.13±6.48^a^ | 27.95±2.36^a^ | 38.71±5.24^b^ | 28.31±1.82^a^ |
| D-BIL(μmol/L) | 1.35±0.18^a^ | 1.65±0.13^b^ | 1.43±0.09^a^ | 1.41±0.16^a^ | 1.75±0.16^b^ | 1.51±0.10^a^ | 1.46±0.13^a^ | 1.79±0.09^b^ | 1.55±0.10^a^ |
| I-BIL (μmol/L) | 23.63±3.57^a^ | 27.69±3.86^a^ | 23.84±2.68^a^ | 25.32±4.44^a^ | 31.15±4.96^a^ | 24.62±6.46^a^ | 26.49±2.40^a^ | 36.92±5.17^b^ | 26.75±1.86^a^ |
| CREA-S(mmol/L) | 15.34±1.31^a^ | 17.24±1.70^a^ | 15.55±1.47^a^ | 14.33±1.04^a^ | 17.16±1.05^b^ | 15.31±1.99^ab^ | 15.07±2.22^a^ | 18.08±1.25^b^ | 15.45±1.89^a^ |
| UA(μmol/L) | 180.57±14.70^a^ | 191.51±17.45^a^ | 186.80±15.87^a^ | 184.07±14.69^a^ | 213.05±25.63^b^ | 187.81±18.87^a^ | 186.30±13.87^a^ | 223.71±26.82^b^ | 187.05±25.63^a^ |
| LDH(U/L) | 965.34±58.38^a^ | 1063.10±96.24^a^ | 973.88±77.67^a^ | 968.14±55.09^a^ | 1137.50±75.21^b^ | 960.63±84.50^a^ | 950.03±63.45^a^ | 1214.88±50.57^b^ | 957.57±164.31^a^ |
| CK(U/L) | 2035.68±260.22^a^ | 2268.80±210.54^a^ | 2026.34±212.41^a^ | 2073.53±183.79^a^ | 2371.73±151.93^b^ | 2135.96±188.91^ab^ | 2057.54±213.50^a^ | 2568.90±196.46^b^ | 2241.04±312.49^ab^ |
| CK-MB(U/L) | 1366.50±128.15^a^ | 1515.60±197.79^a^ | 1359.73±172.37^a^ | 1325.56±152.60^a^ | 1524.31±133.56^b^ | 1343.23±110.29^a^ | 1390±144.96^a^ | 1720.65±131.06^b^ | 1415.93±161.72^a^ |
| T-AOC(U/ml) | 6.89±0.31^b^ | 5.36±0.30^a^ | 6.66±0.24^b^ | 6.45±0.69^b^ | 5.62±0.20^a^ | 6.16±0.27^ab^ | 6.21±0.45^b^ | 5.08±0.45^a^ | 5.59±0.49^b^ |
| SOD(U/ml) | 355.97±34.22^b^ | 261.14±16.11^a^ | 336.20±28.31^b^ | 352.64±37.09^b^ | 258.73±27.92^a^ | 344.86±29.09^b^ | 369.75±27.11^b^ | 267.83±14.08^a^ | 351.17±29.79^b^ |
| NO(μmol/ml) | 60.25±6.61^b^ | 45.19±3.92^a^ | 56.99±3.16^b^ | 61.24±3.83^c^ | 43.24±4.66^a^ | 53.29±3.59^b^ | 64.23±3.39^c^ | 43.49±5.42^a^ | 54.52±6.16^b^ |
| MDA(nmol/ml) | 4.69±0.53^a^ | 5.66±0.34^b^ | 4.86±0.27^a^ | 4.45±0.30^a^ | 5.53±0.32^b^ | 4.77±0.42^a^ | 4.75±0.73^a^ | 5.83±0.54^b^ | 5.05±0.65^ab^ |
| GSH-Px(U/ml) | 463.80±43.33^a^ | 411.33±53.35^a^ | 446.16±34.91^a^ | 468.42±46.08^b^ | 403.21±48.23^a^ | 455.82±32.24 ^ab^ | 459.20±43.14^b^ | 388.62±28.32^a^ | 441.67±48.95^ab^ |

**Data are means of 6 replications with 10 hens/replicate. TP, total protein; ALB, albumin; GLO, globulin; TBIL, total bilirubin; D-BIL, direct bilirubin; I-BIL, indirect bilirubin; CREA-S, creatinine; UA, uric acid; TC, total cholesterol; TG, triglycerides; HDL, high density lipoprotein; LDL, low density lipoprotein; ALT, Alanine transaminase; AST, aspartate transaminase; ALP,** **alkaline phosphatase; γ-GT, γ-glutamyl transferase; LDH,** **lactate dehydrogenase; CK,** **creatine kinase, CK-MB, myocardial creatine kinase; T-AOC, total antioxidative capacity; SOD, superoxide dismutase; NO, nitric oxide; MDA, Malondialdehyde; GSH-Px, glutathione peroxidase.**

| **Table S3.Data preprocessing statistics and quality control.** | | | | |
| --- | --- | --- | --- | --- |
| Sample  Name | Raw  PE^1^ | Clean  Tags^2^ | Clean Tag ^3^ | Q30^4^ |
| TnD07S1 | 75142 | 74980 | 253/0 | 99.78 |
| TnD07S2 | 75234 | 74961 | 252/1 | 99.64 |
| TnD07S3 | 75154 | 74963 | 252/1 | 99.75 |
| TnD07S4 | 75197 | 74936 | 252/1 | 99.65 |
| TnD07S5 | 75186 | 74879 | 252/1 | 99.59 |
| TnD07S6 | 75235 | 74920 | 253/0 | 99.58 |
| TnD14S1 | 75197 | 74896 | 253/0 | 99.6 |
| TnD14S2 | 75127 | 74932 | 252/0 | 99.74 |
| TnD14S3 | 75204 | 74991 | 252/1 | 99.72 |
| TnD14S4 | 75156 | 74981 | 252/1 | 99.77 |
| TnD14S5 | 75112 | 74980 | 252/1 | 99.82 |
| TnD14S6 | 75168 | 74900 | 252/1 | 99.64 |
| TnD21S1 | 75202 | 74910 | 253/0 | 99.61 |
| TnD21S2 | 75220 | 74939 | 252/1 | 99.63 |
| TnD21S3 | 75195 | 74976 | 252/1 | 99.71 |
| TnD21S4 | 75137 | 74909 | 253/0 | 99.7 |
| TnD21S5 | 75216 | 74918 | 252/1 | 99.6 |
| TnD21S6 | 75245 | 74940 | 253/0 | 99.59 |
| PfD07S1 | 75114 | 74905 | 252/1 | 99.72 |
| PfD07S2 | 75180 | 74816 | 252/1 | 99.52 |
| PfD07S3 | 75143 | 74957 | 252/0 | 99.75 |
| PfD07S4 | 75153 | 75022 | 252/1 | 99.83 |
| PfD07S5 | 75164 | 74961 | 252/1 | 99.73 |
| PfD07S6 | 75210 | 74872 | 252/0 | 99.55 |
| PfD14S1 | 75240 | 74961 | 252/0 | 99.63 |
| PfD14S2 | 75144 | 74956 | 252/1 | 99.75 |
| PfD14S3 | 75228 | 74891 | 252/1 | 99.55 |
| PfD14S4 | 75176 | 74920 | 252/1 | 99.66 |
| PfD14S5 | 75173 | 74946 | 252/1 | 99.7 |
| PfD14S6 | 75144 | 74946 | 253/0 | 99.74 |
| PfD21S1 | 75178 | 74961 | 252/0 | 99.71 |
| PfD21S2 | 75239 | 74882 | 252/1 | 99.53 |
| PfD21S3 | 75206 | 74842 | 253/0 | 99.52 |
| PfD21S4 | 75123 | 74956 | 253/0 | 99.78 |
| PfD21S5 | 75166 | 74925 | 252/1 | 99.68 |
| PfD21S6 | 75162 | 74942 | 252/1 | 99.71 |
| HsD07S1 | 75175 | 74983 | 252/1 | 99.74 |
| HsD07S2 | 75223 | 74915 | 252/1 | 99.59 |
| HsD07S3 | 75126 | 74947 | 252/1 | 99.76 |
| HsD07S4 | 75197 | 74816 | 252/1 | 99.49 |
| HsD07S5 | 75119 | 75007 | 252/1 | 99.85 |
| HsD07S6 | 75132 | 74901 | 252/1 | 99.69 |
| HsD14S1 | 75204 | 75028 | 252/1 | 99.77 |
| HsD14S2 | 75200 | 75008 | 253/0 | 99.74 |
| HsD14S3 | 75120 | 74961 | 252/1 | 99.79 |
| HsD14S4 | 75189 | 74817 | 253/0 | 99.51 |
| HsD14S5 | 75108 | 75008 | 252/1 | 99.87 |
| HsD14S6 | 75137 | 74948 | 252/1 | 99.75 |
| HsD21S1 | 75213 | 74880 | 253/0 | 99.56 |
| HsD21S2 | 75219 | 74843 | 252/1 | 99.5 |
| HsD21S3 | 75176 | 74858 | 252/1 | 99.58 |
| HsD21S4 | 75235 | 74745 | 252/1 | 99.35 |
| HsD21S5 | 75220 | 74929 | 253/0 | 99.61 |
| HsD21S6 | 75139 | 74910 | 252/1 | 99.7 |

^1^Raw PE means the original paired end reads off the computer.

^2^Raw Tags refers to splice sequence Tags.

^3^Clean Tag is pointing to the tags that have filtered the low quality and short length sequence.

^4^The AvgLen/SD refers to the average length of the Effective Tags and Standard Deviation.

| **Table S4 Identification of Different Metabolites from Any Two Groups of TN, HS, and PF in Serum^a^** | | | | | | | | | | |
| --- | --- | --- | --- | --- | --- | --- | --- | --- | --- | --- |
| **Ionization** | **Metabolite** | **Rt(s)** | **m/z** | **HS vs TN** | | **PF vs TN** | | **HS vs PF** | | **Metabolic pathway** |
|  |  |  |  | **Log2FC** | **Trend** | **Log2FC** | **Trend** | **Log2FC** | **Trend** |  |
| POS | 4-Aminobutyric acid | 37.80 | 224.16 | -0.36 | ↓ | 1.50 | ↑^**^ | -1.88 | ↓^**^ | alanine, aspartate and glutamate metabolism |
| Neg | L-Asparagine | 378.60 | 131.04 | 2.26 | ↑^**^ | 1.53 | ↑^**^ | 0.73 | ↑^*^ |  |
| POS | Ornithine | 189.03 | 155.08 | 2.73 | ↑^**^ | 1.50 | ↑^**^ | 1.23 | ↑^**^ | arginine and proline metabolism |
| POS | Hydroxyproline | 409.50 | 132.07 | 1.70 | ↑^**^ | 1.28 | ↑^*^ | 0.43 | ↑ |  |
| POS | Creatinine | 162.27 | 114.07 | 1.71 | ↑^**^ | 1.06 | ↑^*^ | 0.65 | ↑^*^ |  |
| Neg | Urea | 23.87 | 65.99 | 1.72 | ↑^*^ | 1.53 | ↑^*^ | 0.21 | ↑ |  |
| POS | N-Methylhydantoin | 43.18 | 246.12 | -2.01 | ↓^**^ | -0.60 | ↓ | -1.43 | ↓^**^ |  |
| Neg | Putrescine | 331.39 | 88.04 | -1.64 | ↓^**^ | -0.11 | ↓ | -1.67 | ↓^**^ |  |
| POS | L-Methionine | 269.52 | 150.06 | 1.41 | ↑^**^ | -0.45 | ↓ | 1.87 | ↑^**^ | cysteine and methionine metabolism |
| Neg | Cysteine | 37.36 | 121.03 | 1.69 | ↑^*^ | 0.01 | ↑ | 1.68 | ↑^*^ |  |
| POS | α-Aminoadipic acid | 338.60 | 161.61 | -1.65 | ↓^*^ | -0.62 | ↓ | -1.08 | ↓ | lysine degradation |
| POS | Glycine | 347.37 | 117.07 | 1.18 | ↑^*^ | -0.61 | ↓ | 1.80 | ↑^**^ | glycine, serine and threonine metabolism |
| POS | L-Tyrosine | 323.88 | 182.08 | 1.84 | ↑^**^ | 1.06 | ↑^*^ | 0.79 | ↑^*^ | phenylalanine and tyrosine metabolism |
| POS | L-Phenylalanine | 243.80 | 166.09 | 1.21 | ↑^**^ | 0.92 | ↑^*^ | 0.29 | ↑ |  |
| POS | L-Glutamine | 417.87 | 147.08 | 1.58 | ↑^**^ | 1.13 | ↑^*^ | 0.46 | ↑ | D-glutamine and D-glutamate metabolism |
| POS | Arachidonic Acid | 32.44 | 269.23 | -1.35 | ↓ | 1.30 | ↑^*^ | -2.71 | ↓^**^ | fatty acid metabolism |
| POS | Heptanoic acid | 25.77 | 130.17 | -1.41 | ↓ | 1.98 | ↑^*^ | -3.59 | ↓^**^ |  |
| POS | Stearic acid | 47.54 | 302.30 | -1.13 | ↓^*^ | 1.26 | ↑^**^ | -2.41 | ↓^**^ |  |
| POS | Palmitic acid | 47.44 | 274.27 | -1.16 | ↓^*^ | 1.05 | ↑^**^ | -2.22 | **↓^*^** |  |
| POS | Oleic acid | 38.58 | 324.29 | -1.86 | ↓^*^ | 1.36 | ↑^*^ | -3.30 | ↓^**^ |  |
| POS | Myristic acid | 47.74 | 246.24 | -2.14 | ↓^**^ | 1.31 | ↑^**^ | -3.48 | ↓^**^ |  |
| Neg | Linoleic acid | 41.98 | 559.47 | -0.12 | ↓ | 1.70 | ↑^**^ | -1.67 | ↓^**^ |  |
| Neg | Glycerol | 23.10 | 92.99 | 0.15 | ↑ | 1.78 | ↑^**^ | -1.63 | ↓^**^ | glycerolipid metabolism |
| Neg | Glyceric acid | 43.21 | 87.01 | -1.64 | ↓^*^ | -1.64 | ↓^**^ | -0.003 | ↓ |  |
| POS | 7-Dehydrocholesterol | 38.06 | 385.3 | 1.58 | ↑^**^ | -2.20 | ↓^**^ | 3.83 | ↑^**^ | steroid biosynthesis |
| POS | (R)-3-Hydroxybutyric acid | 76.50 | 165.08 | -2.18 | ↓^**^ | 1.24 | ↑^**^ | -3.49 | ↓^**^ | Synthesis and degradation of ketone bodies |
| Neg | D-Fructose | 292.45 | 239.07 | 1.78 | ↑^**^ | 1.67 | ↑^**^ | 0.11 | ↑ | glycolysis |
| Neg | Glycerol 3-phosphate | 371.92 | 171.00 | -1.66 | ↓^*^ | 1.70 | ↑^**^ | -3.36 | ↓^**^ |  |
| Neg | D-glucose | 302.34 | 179.09 | -2.49 | ↓^**^ | -1.58 | ↓^**^ | -0.91 | ↓ |  |
| Neg | Pyruvate | 87.007 | 130.32 | 1.15 | ↑^**^ | -0.84 | ↓ | 2.00 | ↑^**^ |  |
| Neg | Myo-Inositol | 378.41 | 179.05 | -1.65 | ↓^**^ | 1.66 | ↑^**^ | -3.29 | ↓^**^ | inositol phosphate metabolism |
| POS | D-Xylitol | 234.13 | 175.06 | -0.41 | ↓ | -2.46 | ↓^**^ | 2.07 | ↑^**^ | Pentose and glucuronate interconversions |
| POS | Succinic acid | 260.11 | 117.72 | 1.59 | ↑^**^ | 1.90 | ↑^**^ | -0.30 | ↓ | TCA cycle |
| POS | Citric acid | 33.61 | 192.14 | 1.03 | ↑^*^ | 1.36 | ↑^**^ | -0.33 | ↓ |  |
| Neg | Fumaric acid | 379.58 | 116.03 | 1.74 | ↑^**^ | 2.42 | ↑^**^ | -0.68 | ↓^*^ |  |
| Neg | α-Ketoglutaric acid | 500.04 | 145.10 | 1.73 | ↑^**^ | 2.48 | ↑^**^ | -0.73 | ↓^*^ |  |
| Neg | Aconitic acid | 428.07 | 173.01 | 1.78 | ↑^**^ | 2.53 | ↑^**^ | -0.75 | ↓^*^ |  |
| Neg | Uric acid | 317.03 | 167.02 | 1.96 | ↑^**^ | 0.04 | ↑ | 1.92 | ↑^**^ | purine metabolism |
| Neg | D-Ribose | 324.48 | 149.04 | 0.19 | ↑ | 1.82 | ↑^**^ | -1.63 | ↓^**^ |  |
| POS | Uracil | 156.37 | 113.03 | 0.95 | ↑ | 1.35 | ↑^**^ | -0.39 | ↓ | pyrimidine metabolism |
| Neg | Thymine | 141.32 | 125.03 | 1.89 | ↑^**^ | 1.62 | ↑^**^ | 0.28 | ↑ |  |
| POS | Methylmalonic acid | 162.15 | 119.03 | 1.37 | ↑^*^ | -0.60 | ↓ | 2.01 | ↑^**^ |  |
| POS | Nicotinamide | 62.98 | 123.06 | 0.83 | ↑^*^ | 1.07 | ↑^**^ | -0.25 | ↓ | nicotinate and nicotinamide metabolism |
| POS | γ-Tocopherol | 250.55 | 416.26 | -2.20 | ↓^**^ | -0.64 | ↓ | -1.59 | ↓^**^ | vitamin E metabolism |
| POS | L-Citrulline | 305.24 | 242.05 | -2.13 | ↓^**^ | -0.56 | ↓ | -1.63 | ↓^*^ | other |
| POS | L-Carnitine | 338.60 | 162.11 | -0.55 | ↓^*^ | -0.68 | ↓^*^ | 0.12 | ↑ |  |
| POS | Glutaraldehyde | 286.81 | 123.04 | 1.42 | ↑^**^ | 1.34 | ↑^**^ | 0.09 | ↑ |  |
| POS | Acetylcarnitine | 292.03 | 204.12 | -0.83 | ↓^*^ | -0.52 | ↓ | -0.31 | ↓ |  |
| Neg | Ketoisocaproic acid | 44.56 | 129.05 | 0.11 | ↑ | -1.64 | ↓^**^ | 1.75 | ↑^**^ |  |

**^a^↓: downregulation. ↑: upregulation. FC: fold change. VIP:variable importance in the projection. Data were analyzed by t test (n = 5). *p < 0.05, **p < 0.01.**


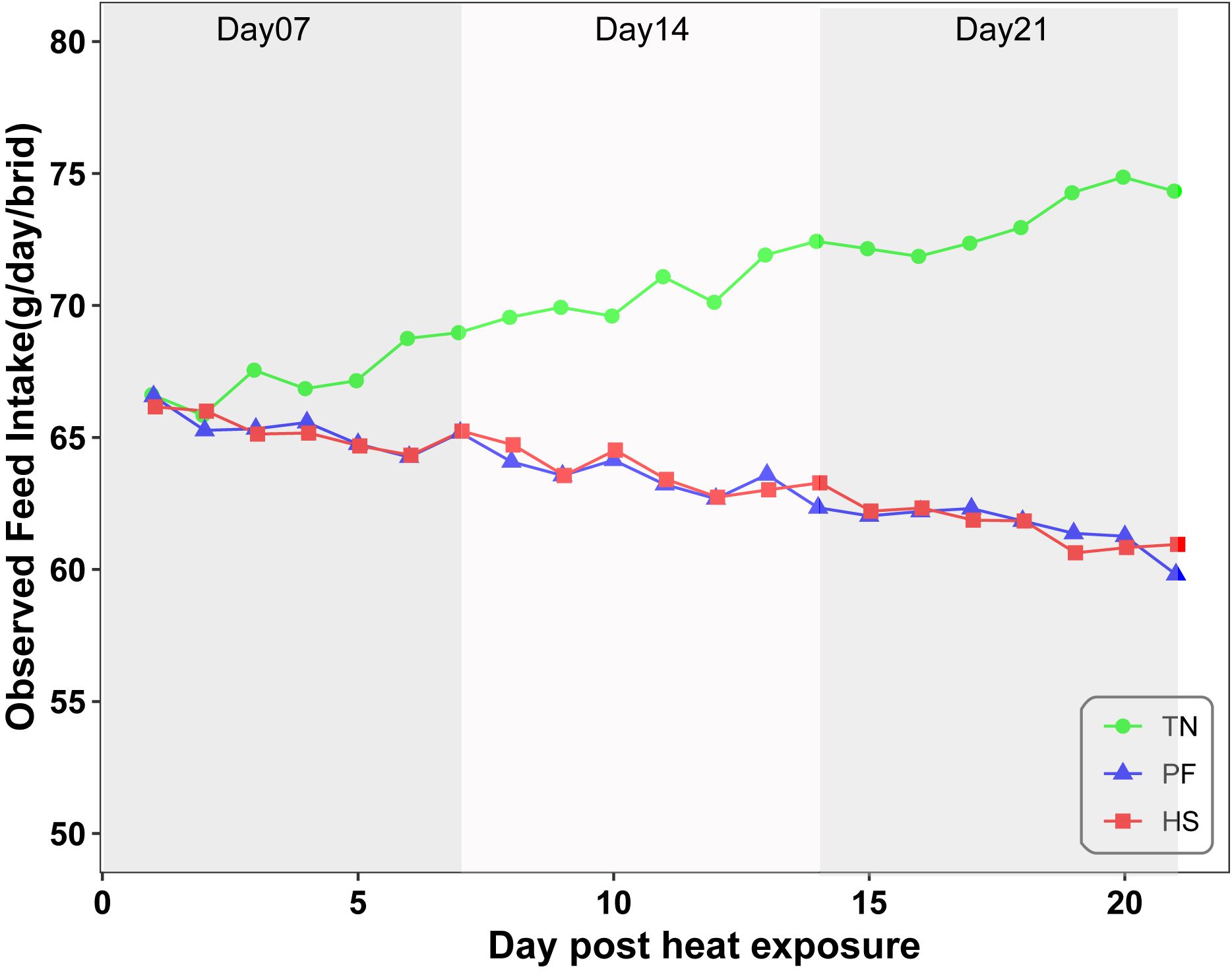


Fig. S1. Graphs showing the observed feed intake of the three groups during the experimental period.


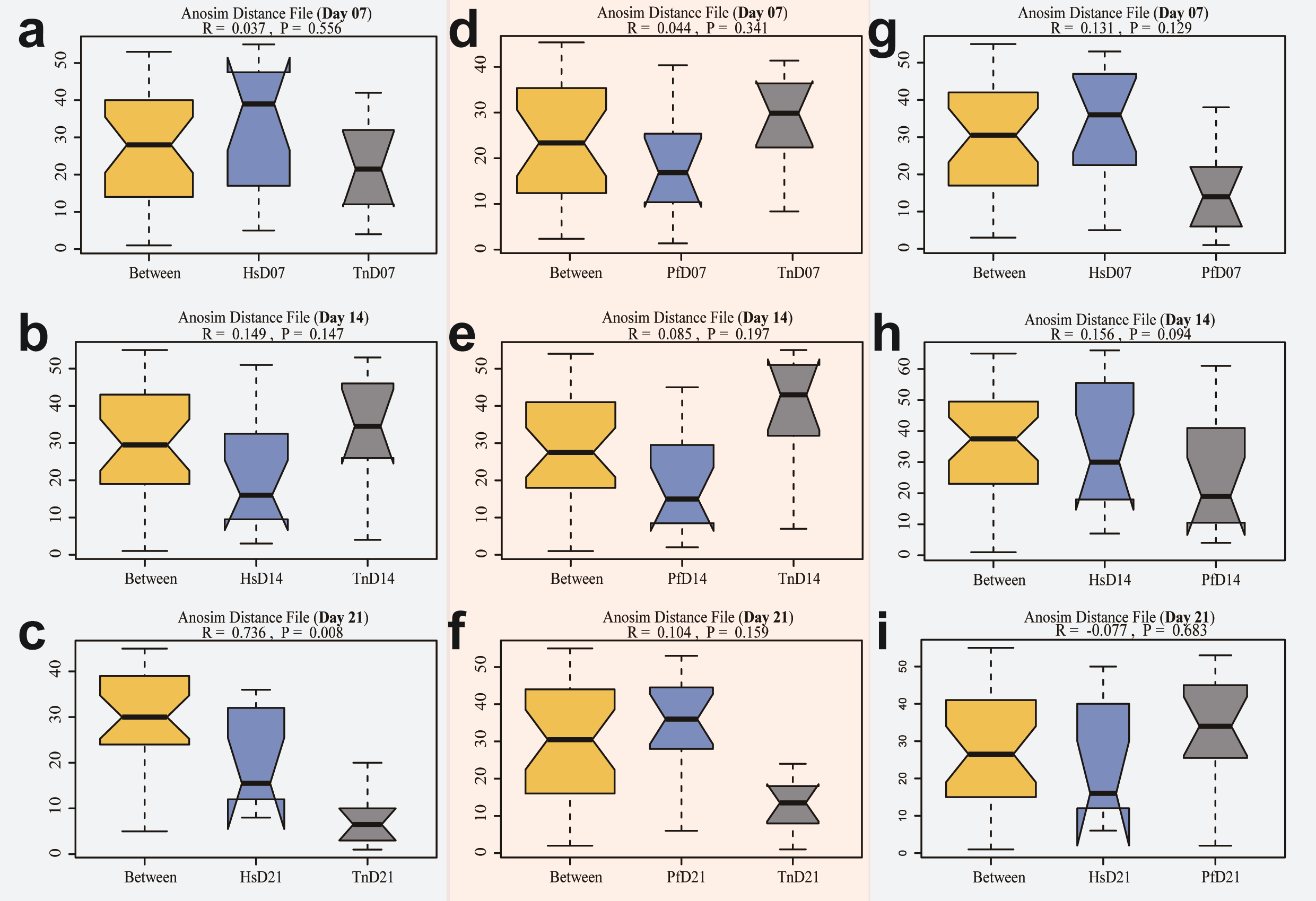


Fig. S2. Plot of analysis of similarities (ANOSIM) based on the unweighted UniFrac distance matrixes. (a-b) TN vs. HS; (d-f) TN vs. PF; (g-i) PF vs. HS.


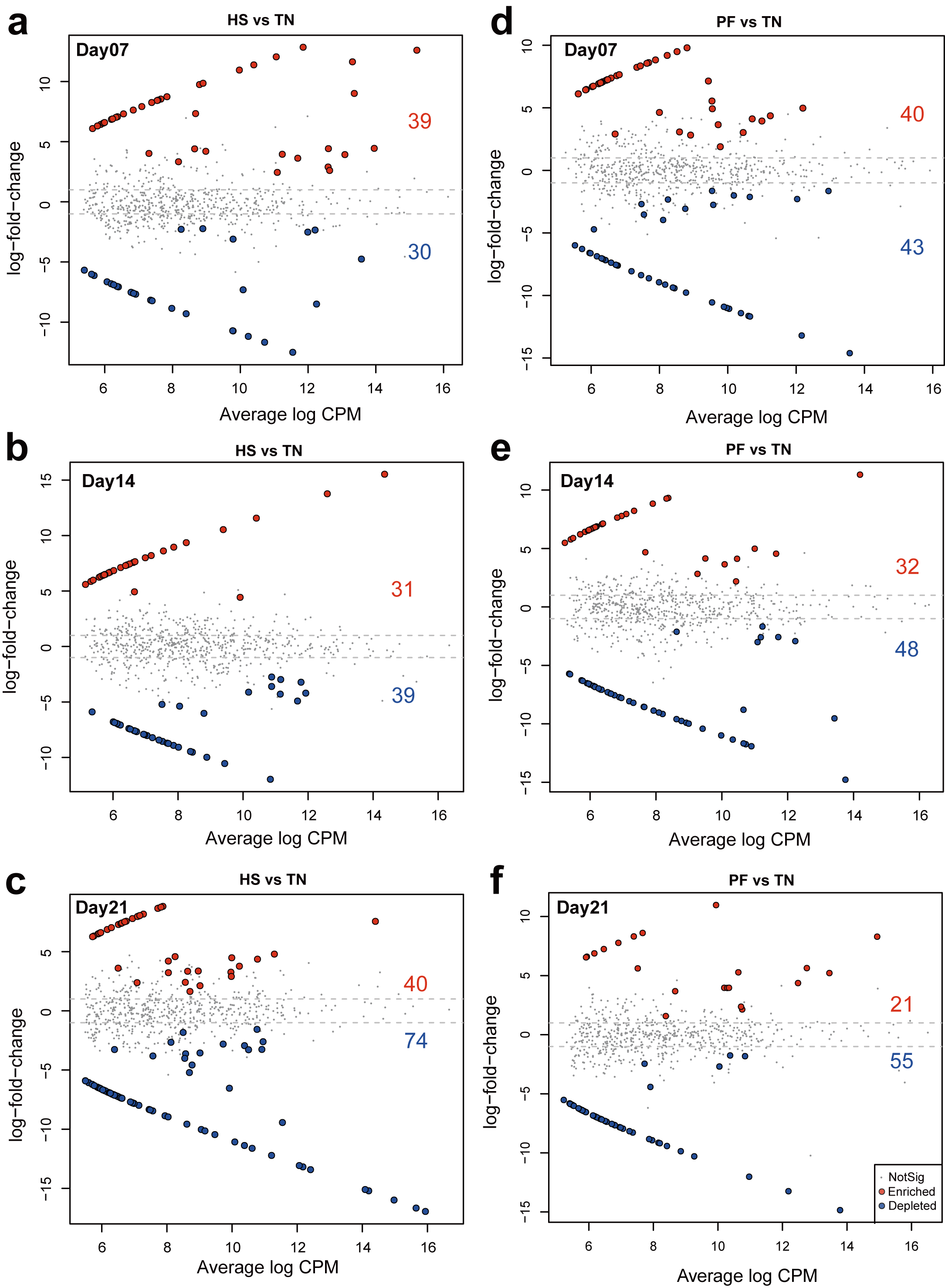


Fig. S3. MA plot for the OTUs changes among the three experimental groups. (a-b) TN vs. HS; (d-f) TN vs. PF.


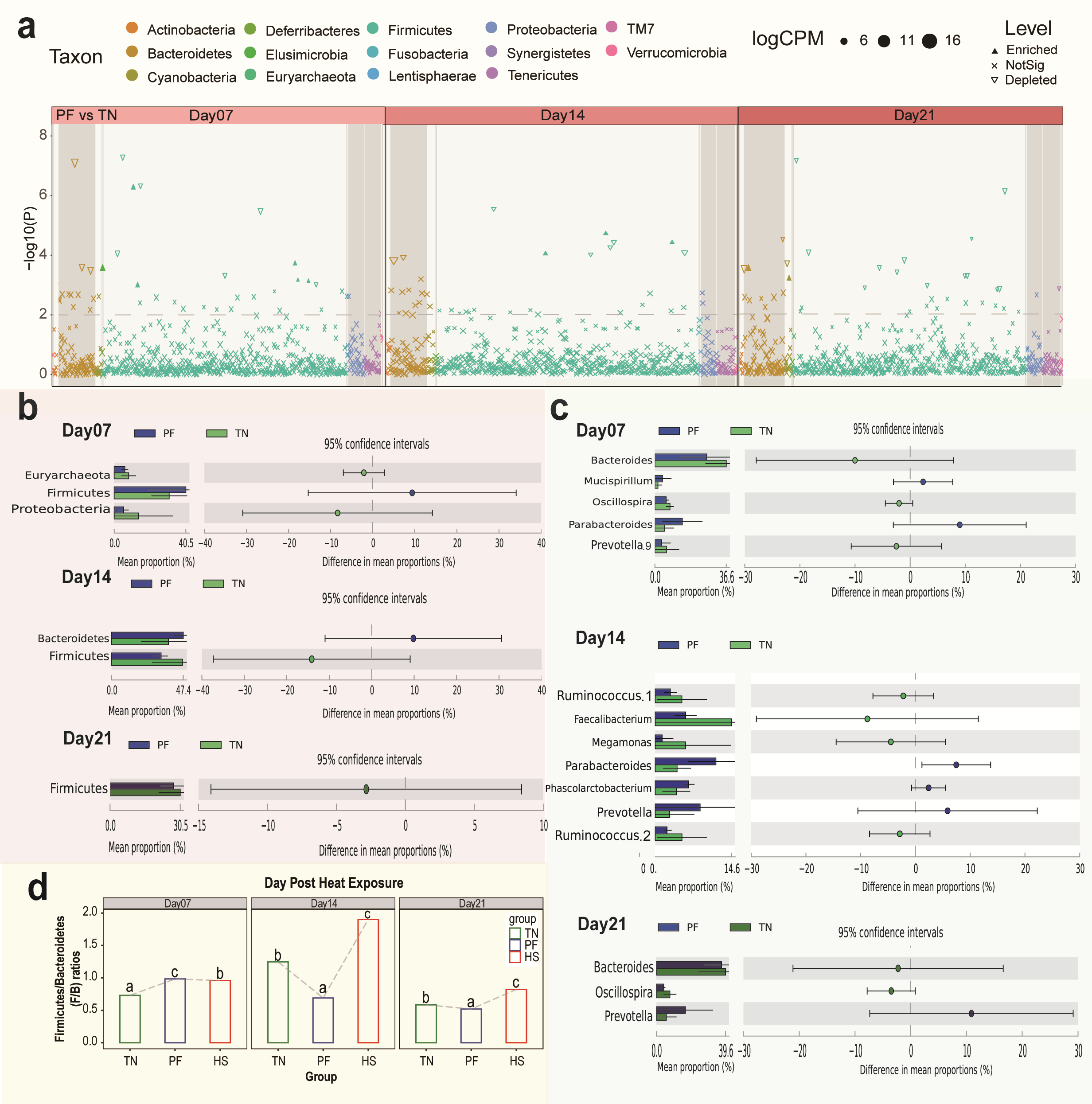


Fig. S4. Effects of chronic heat exposure on cecal microbial composition at different taxonomic levels in growing hens. (a) The manhattan plots show enrichment and depletion of microbial OTUs between PF and TN group during the experimental period (on day 7, 14 and 21). The dashed line represents the significant level (using false-discovery rate-corrected P values of 0.01). A statistical comparison between differences in the relative abundance of dominant microbial phyla (b) and genera (c) among three experimental groups based on Welch’s t test (P < 0.05, effect sizes>2). (d) Bar plots showing the ratio of F/B among the TN, PF and HS groups. All results are expressed as mean ± SD of eight hens in each group. Means with the same letter (a, b, c) do not differ statistically; means with different letter are statistically different (p <0.05).


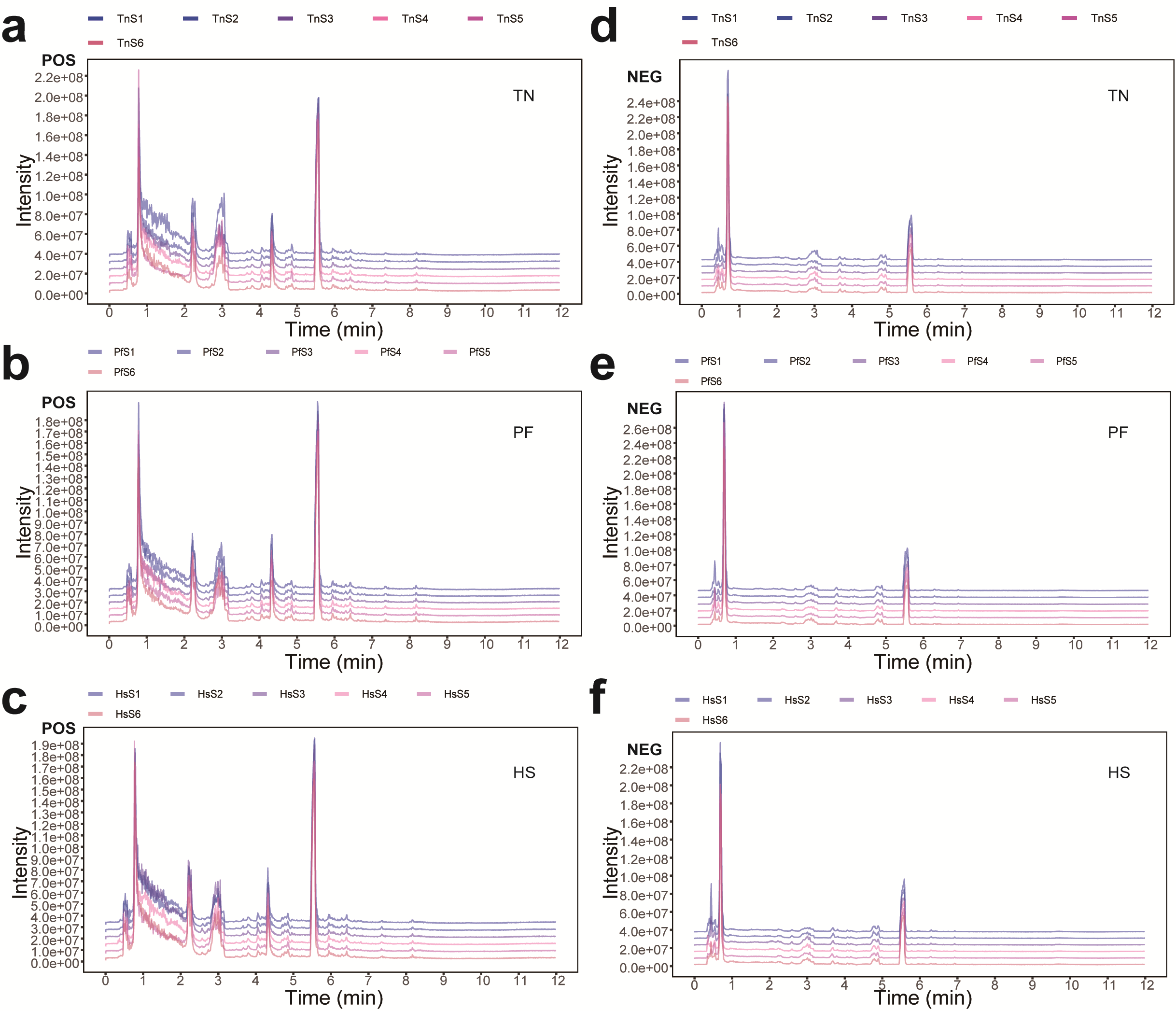


Fig. S5. Representative UHPLC-QTOF/MS total ion chromatograms (TIC) of serum got from the TN group (a & d), PF group (b & e), and HS group (c & f) analyzed in positive and negative-ion modes, respectively.


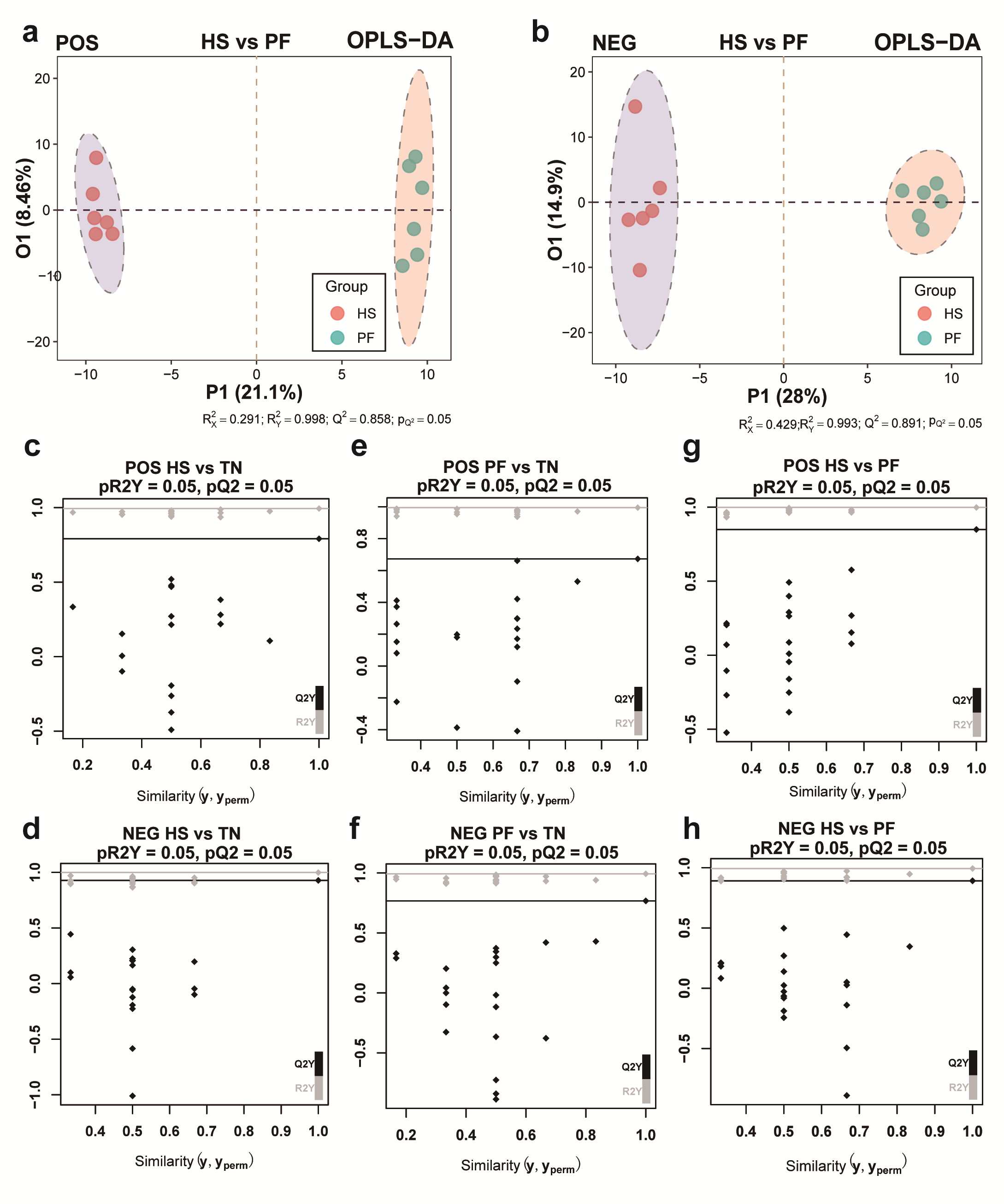


Fig. S6. OPLS-DA score chart of serum metabolite analysis the variance of positive (a) and negative (b) ions between the HS and PF groups. Permutation test of the OPLS-DA model (TN group vs. HS group) in positive (c) and negative (d) ions model. Validation plots of the OPLS-DA models (PF group vs. TN group) acquired through 999 permutation tests in positive (e) and negative (f) ions model. Permutation test of the OPLS-DA model (HS group vs. PF group) in positive (g) and negative (h) ions model.


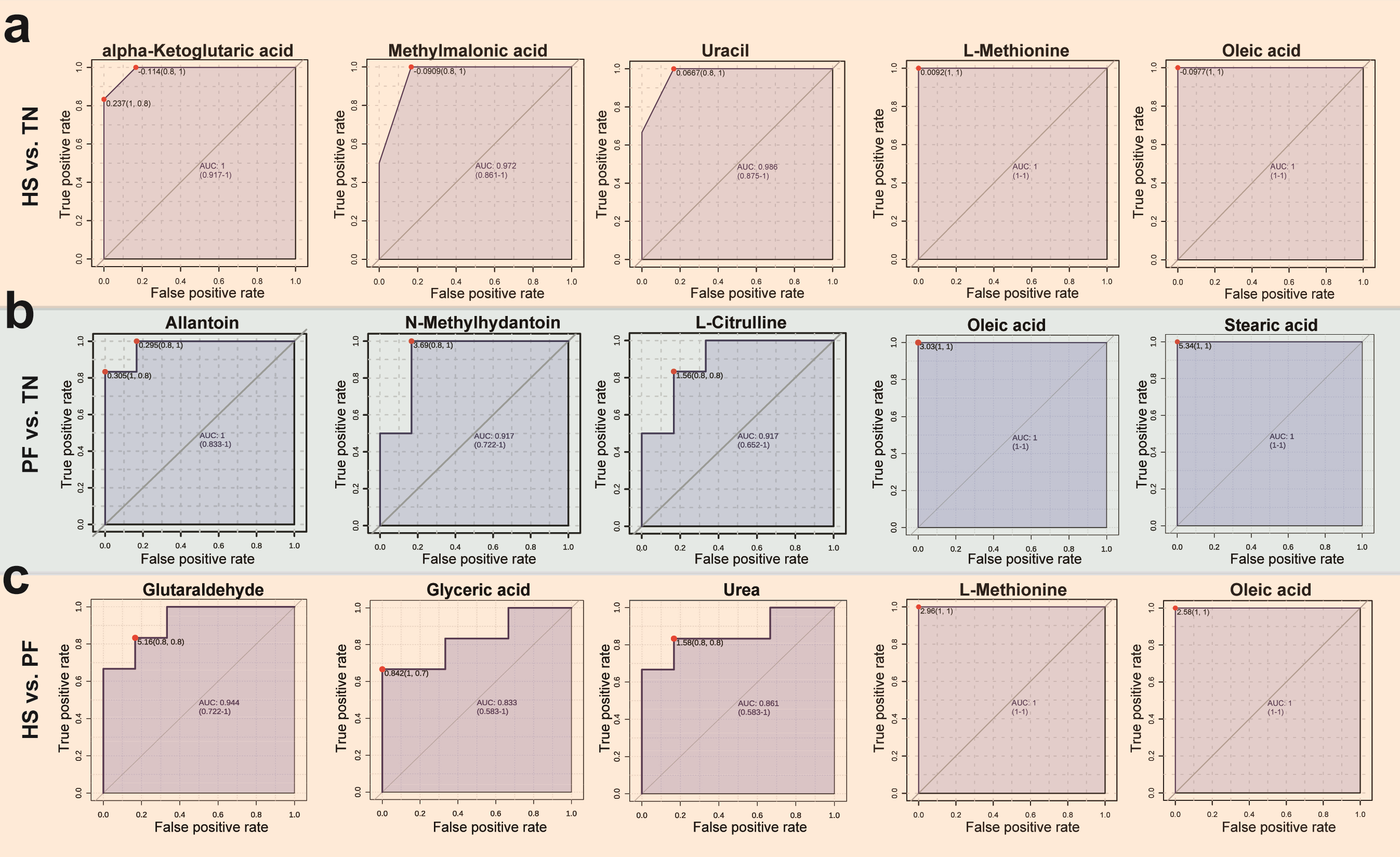


Fig. S7. ROC curve analysis of differential metabolites between different groups. (a) The comparison between the HS and TN groups. (b) The comparison between the PF and TN groups. (c) The comparison between the HS and PF groups.


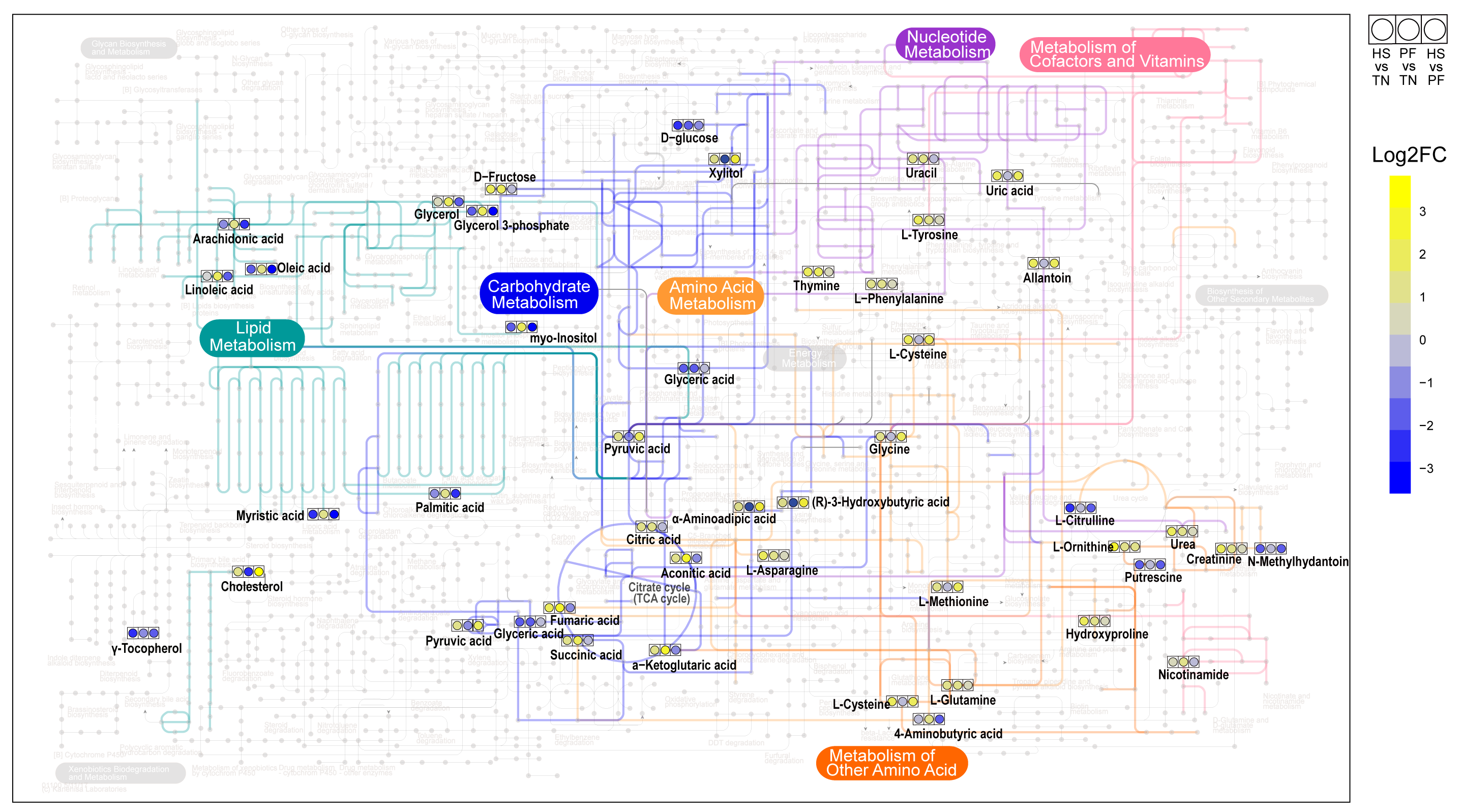


Fig. S8. Different metabolic pathways from the three experimental groups. The color gradients on the right show the value of Log2 fold change between different groups.

**DNA extraction and quantitative PCR amplification**

Total microbial genomic DNA, including bacterial and fungal genomic DNA in the cecal content of chickens, were extracted using a combined method of cetyltrimethyl ammonium bromide/sodium dodecyl sulfate (CTAB/SDS) method. Briefly, the concentration and purity of DNA were tested with 1% agarose gel, and the DNA were diluted with sterile water to 1 ng/μl. Extracted metagenomic DNA were stored at－20 °C for subsequent analysis.

The V3–V4 hyper-variable regions of the 16 S rDNA gene were amplified by specific degenerate primers (515F 5′-GTGCCAGCMGCCGCGGTAA-3′ and 806R 5′-GGACTACHVGGGTWTCTAAT-3′; M stands for A/C, V stands for A/C/G, and S stands for C/G)) with unique barcodes. All PCR reactions were carried out in 30 μL reactions with 15 μL of Phusion® High-Fidelity PCR Master Mix (New England Biolabs); 0.2 μM of forward and reverse primers, and about 10 ng template DNA. Thermal cycling consisted of initial denaturation at 98 °C for 1 min, followed by 30 cycles of denaturation at 98 °C for 10s, annealing 50 °C for 30 s, and elongation at 72 °C for 30s, and a finally extension at 72 °C for 5 min. Samples with a bright main strip between 400 and 450 bp after electrophoresis on 2% agarose gel were selected for further experiments. **Illumina HiSeq sequencing analysis**

PCR products were mixed in equidensity ratios. The mixture of PCR products were purified with GeneJET Gel Extraction Kit (Thermo Scientific). The libraries were sequenced on Illumina HiSeq platform (Illumina, Inc., San Diego, CA, USA) and 250 bp paired-end reads were generated at BGI Co., Ltd (Wuhan, China). Paired-end reads were merged using FLASH (ver. 1.2.7, http://ccb.jhu.edu/software/FLASH/) and the splicing sequences were raw tags. For quality-filtering of raw data, the sequences shorter than 200 bp or longer than 470 bp and sequences containing primer mismatches, uncorrectable barcodes, ambiguous bases, or homopolymer runs in excess of six bases were discarded. The sequences that passed the quality filters were analysed using the open-source software system QIIME (Quantitative Insights into Microbial Ecology) (Caporaso et al., 2010a). The sequences were assigned operational taxonomic units (OTUs) using the reference with de novo based approach using UCLUST(Edgar, 2010) at 97% identity against the Greengenes database (version 13.5) (DeSantis et al., 2006). All the raw sequence data in the present study were deposited in the National Center for Biotechnology Information (NCBI) under the accession number PRJNA797199.

All steps of sequence analysis were performed using QIIME and R packages (version 3.2.0) (Kuczynski et al., 2011). In order to evaluate individual hosts' microbial alpha diversity, an OTU table including Good's coverage was produced and the rarefaction curve was generated based on the metric of observed species (Bartram et al., 2011). The alpha diversity indices, including Chao1 (which measures richness based on rare OTUs) and Shannon (which measures richness and evenness), were calculated to reflect the diversity and richness of the community in different samples (Chao and Lee, 1992; Schloss et al., 2005). The beta diversity, which describes the species differences among different samples, was calculated using weighted Bray-Curtis distance matrix and visualized with principle coordinates analysis (PCoA). The same QIIME software calculated the jaccard distance and created the unweighted pair-group method with arithmetic means (UPGMA) clustering tree (Caporaso et al., 2010b). The classification comparing graphs at various levels of species composition analysis were produced using QIIME software. Wilcox test was used for calculating differences in these microbial taxa among the three groups at the phylum level and genus level. STAMP was used to compare cecal microbial composition between three groups (Ge et al., 2021; Parks et al., 2014), and the default Welch's t-test was chosen at p level of 0.05 and effect size of 2. Phylogenetic investigation of communities by reconstruction of unobserved states (PICRUSt) uses an extended ancestral-state reconstruction algorithm to predict which gene families are present and then combines gene families to estimate the composite metagenome (Langille et al., 2013). The predicted genes and their respective functions were aligned to the Kyoto Encyclopedia of Genes and Genomes (KEGG) database, and the differences between the two groups were examined in STAMP software (http://kiwi.cs.dal ca/ Software/STAMP)(Kanehisa and Goto, 2000).

**Serum sample preparation**

The serum samples were prepared as with the following procedure. First, 300 μL of methanol and 20 μL of L-2-chlorophenylalanine (internal standard) were added to a 100 μL sample, mixed by vortexing 30 s, and sonicated for 10 min in an ice-water bath. The solution was then incubated at - 20 °C for 1 h, and centrifuged at 4 °C and 12,000 rpm for 15 min. Subsequently, 200 μL of supernatant was placed into a 2-mL glass vial pending for detection. Equal volumes (20 μL) of supernatant from different individual serum samples were pooled as the quality control (QC) sample for the UHPLC-QTOF/MS analysis. The QC samples (n = 10) were used to assess reproducibility and reliability of the LC-MS system.

**UHPLC-QTOF/MS conditions**

Serum metabolic profiling analysis was performed on a 1290 UHPLC system (Agilent Technologies, Santa Clara, CA, USA) with a Waters ACQUITY UPLC BEH C18 column (2.1 mm × 100 mm, 1.7 µm) coupled to a triple time-of-flight 6600 (Q-TOF, AB Sciex, Framingham, MA, USA). The mobile phase for chromatographic separation was composed of (A) deionized water containing 25 mM ammonium acetate and 25 mM NH_4_OH (pH = 9.75) and (B) acetonitrile with gradient elution as follows: 0–0.5 min, A: 5%; 0.5–7 min, A: 5–35%; 7–8 min, A: 35–60%; 8–9 min, A: 60%; 9–9.1 min, A: 60–5%; 9.1–12 min, A: 5%. The flow rate was 0.5 mL/min, the column temperature was maintained at 35℃, and 5μL was injected into the system. The mass spectroscopy (MS) data were collected from m/z 50–1200 Da with an electrospray ionization (ESI) source in positive and negative ion modes. MS spectra acquisition was performed using Analyst TF 1.7 software (AB Sciex) based on information-dependent basis (IDA) function. In each cycle, the precursor ions with intensity greater than 100 were chosen for fragmentation at collision energy of 30eV (15 MS/MS events per 50ms of product ion accumulation time). The ionization source conditions were as follows: nebulizer pressure, 60 psi; auxiliary pressure, 60 psi; curtain gas, 35 psi; gas temperature, 650 °C; capillary voltage, 5000 V in positive mode, and 4000 V in negative mode.

**UHPLC-QTOF/MS data processing**

MS raw data (.wiff) files from metabolite fingerprinting of UHPLCQTOF/MS were converted to the mzXML format in centroid mode using ProteoWizard and processed by R package XCMS (various forms (X) of chromatography mass spectrometry, version 1.41.0). The data pretreatment of XCMS included untargeted peak detection, peak alignment, peak grouping, retention time (RT) normalization and second peak grouping, and integration on each full data set (experimental study samples and QC samples (Smith et al., 2006). The data matrix of pre-processing results consisted of the RT, mass-to-charge ratio (m/z) values, and peak intensity. Internal standard normalization method was employed to linearly shift the RT across the entire run for metabolite analysis. R package CAMERA (Collection of annotation related methods for mass spectrometry data) was used for the annotation of peak lists generated by XCMS (Kuhl et al., 2012). The in-house MS2 commercial database that included over 1500 MS/MS spectra of metabolites was used to detect MS/MS spectral similarity between compounds for identification of putative metabolites. The data matrix was Ctr-formatted (mean-centered scaling) and Pareto-scaled prior to being imported into the SIMCA-P+ 14.1 software package (Umetrics, Umea, Sweden). The multivariate data analysis (MVDA) included principal components analysis (PCA) and orthogonal partial least squared-discriminant analysis (OPLS-DA). The unsupervised PCA was implemented to demonstrate the distribution of origin data and general separation. The supervised OPLS-DA was performed to obtain maximal covariance between the measured data and the response variable, and validated by 7-fold cross-validation and 200 permutation tests. SPSS 22.0 software (SPSS Inc., Chicago, IL, USA) was utilized for statistical analysis of the normalized integral values to determine significant differences between metabolic changes. Data were analyzed by Student's t-test at significance levels P < 0.05. Those metabolic features with variable importance in projection (VIP) value > 1.0 in the OPLS-DA model and values P < 0.05 in the Student's t-test were considered to be significantly differential metabolites.

**Metabolic pathway analysis**

To gain insight into the biological mechanisms associated with CHE hens, we performed MetPA (www.metaboanalyst.ca) of the differentially expressed metabolites detected in both ion modes using MetaboAnalyst 3.0. It is a free web-based tool that combines results from powerful pathway enrichment analysis with the topology analysis. The pathway library of Gallus gallus (chicken) was selected in the procedure. The hypergeometric test was specified for the over-representation analysis method of the pathway enrichment analysis to test if compounds involved in a particular pathway were enriched compared by random hits. Relative-betweeness centrality was specified for pathway topology analysis. Measuring the number of shortest paths going through the metabolite node helped weigh the metabolite importance on global network topology. For comparison among the different pathways, the node importance values calculated from centrality measures were further normalized by the sum of the importance of the pathway. Therefore, the importance measure of each metabolite node is actually the percentage of the total pathway importance, and the pathway impact value is the cumulative percentage from the matched metabolite nodes (Mitrea et al., 2013). Since the pathway analysis was performed to test many pathways at the same time, the statistical P values from enrichment analysis were further adjusted for multiple testing, in particular, Holm-Bonferroni method and False Discovery Rate (FDR) adjustment.

**References**

Bartram AK, Lynch MD, Stearns JC, Moreno-Hagelsieb G, Neufeld JDJA, microbiology e. Generation of multimillion-sequence 16S rRNA gene libraries from complex microbial communities by assembling paired-end Illumina reads. 2011; 77: 3846-3852.

Caporaso JG, Kuczynski J, Stombaugh J, Bittinger K, Bushman FD, Costello EK, et al. QIIME allows analysis of high-throughput community sequencing data. Nature Methods 2010a; 7: 335-336.

Caporaso JG, Kuczynski J, Stombaugh J, Bittinger K, Bushman FD, Costello EK, et al. QIIME allows analysis of high-throughput community sequencing data. 2010b; 7: 335-336.

Chao A, Lee S-MJJotAsA. Estimating the number of classes via sample coverage. 1992; 87: 210-217.

DeSantis TZ, Hugenholtz P, Larsen N, Rojas M, Brodie EL, Keller K, et al. Greengenes, a chimera-checked 16S rRNA gene database and workbench compatible with ARB. 2006; 72: 5069-5072.

Edgar RCJB. Search and clustering orders of magnitude faster than BLAST. 2010; 26: 2460-2461.

Ge A-H, Liang Z-H, Xiao J-L, Zhang Y, Zeng Q, Xiong C, et al. Microbial assembly and association network in watermelon rhizosphere after soil fumigation for Fusarium wilt control. 2021; 312: 107336.

Kanehisa M, Goto SJNar. KEGG: kyoto encyclopedia of genes and genomes. 2000; 28: 27-30.

Kuczynski J, Stombaugh J, Walters WA, González A, Caporaso JG, Knight RJCpib. Using QIIME to analyze 16S rRNA gene sequences from microbial communities. 2011; 36: 10.7. 1-10.7. 20.

Kuhl C, Tautenhahn R, Bottcher C, Larson TR, Neumann SJAc. CAMERA: an integrated strategy for compound spectra extraction and annotation of liquid chromatography/mass spectrometry data sets. 2012; 84: 283-289.

Langille MG, Zaneveld J, Caporaso JG, McDonald D, Knights D, Reyes JA, et al. Predictive functional profiling of microbial communities using 16S rRNA marker gene sequences. 2013; 31: 814-821.

Mitrea C, Taghavi Z, Bokanizad B, Hanoudi S, Tagett R, Donato M, et al. Methods and approaches in the topology-based analysis of biological pathways. 2013; 4: 278.

Parks DH, Tyson GW, Hugenholtz P, Beiko RGJB. STAMP: statistical analysis of taxonomic and functional profiles. 2014; 30: 3123-3124.

Schloss PD, Handelsman JJA, microbiology e. Introducing DOTUR, a computer program for defining operational taxonomic units and estimating species richness. 2005; 71: 1501-1506.

Smith CA, Want EJ, O'Maille G, Abagyan R, Siuzdak GJAc. XCMS: processing mass spectrometry data for metabolite profiling using nonlinear peak alignment, matching, and identification. 2006; 78: 779-787.
